# Community genomic analyses constrain the distribution of metabolic traits across the Chloroflexi phylum and indicate roles in sediment carbon cycling

**DOI:** 10.1186/2049-2618-1-22

**Published:** 2013-08-05

**Authors:** Laura A Hug, Cindy J Castelle, Kelly C Wrighton, Brian C Thomas, Itai Sharon, Kyle R Frischkorn, Kenneth H Williams, Susannah G Tringe, Jillian F Banfield

**Affiliations:** 1 Department of Earth and Planetary Science, UC Berkeley, Berkeley, CA, USA; 2 Geophysics Department, Earth Sciences Division, Lawrence Berkeley National Lab, Berkeley, CA, USA; 3 Metagenome Program, DOE Joint Genome Institute, Walnut Creek, CA, USA

**Keywords:** Chloroflexi, Metagenome, GIF9, Anaerolinea, Sediment, *Dehalococcoides*, Wood-Ljungdahl, Acetogenesis

## Abstract

**Background:**

Sediments are massive reservoirs of carbon compounds and host a large fraction of microbial life. Microorganisms within terrestrial aquifer sediments control buried organic carbon turnover, degrade organic contaminants, and impact drinking water quality. Recent 16S rRNA gene profiling indicates that members of the bacterial phylum Chloroflexi are common in sediment. Only the role of the class Dehalococcoidia, which degrade halogenated solvents, is well understood. Genomic sampling is available for only six of the approximate 30 Chloroflexi classes, so little is known about the phylogenetic distribution of reductive dehalogenation or about the broader metabolic characteristics of Chloroflexi in sediment.

**Results:**

We used metagenomics to directly evaluate the metabolic potential and diversity of Chloroflexi in aquifer sediments. We sampled genomic sequence from 86 Chloroflexi representing 15 distinct lineages, including members of eight classes previously characterized only by 16S rRNA sequences. Unlike in the Dehalococcoidia, genes for organohalide respiration are rare within the Chloroflexi genomes sampled here. Near-complete genomes were reconstructed for three Chloroflexi. One, a member of an unsequenced lineage in the Anaerolinea, is an aerobe with the potential for respiring diverse carbon compounds. The others represent two genomically unsampled classes sibling to the Dehalococcoidia, and are anaerobes likely involved in sugar and plant-derived-compound degradation to acetate. Both fix CO_2_ via the Wood-Ljungdahl pathway, a pathway not previously documented in Chloroflexi. The genomes each encode unique traits apparently acquired from Archaea, including mechanisms of motility and ATP synthesis.

**Conclusions:**

Chloroflexi in the aquifer sediments are abundant and highly diverse. Genomic analyses provide new evolutionary boundaries for obligate organohalide respiration. We expand the potential roles of Chloroflexi in sediment carbon cycling beyond organohalide respiration to include respiration of sugars, fermentation, CO_2_ fixation, and acetogenesis with ATP formation by substrate-level phosphorylation.

## Background

Sediment environments represent one of the major carbon reservoirs on the planet, and contain a wide variety of uncharacterized microbial lineages [1-5]. Metagenomic sequencing of sediments allows determination of the diversity within complex communities of low-abundance organisms, simultaneously providing genomic sampling of uncultivated organisms and prediction of novel metabolisms and enzymes [1,2]. Recent advances in sequencing technologies provide much greater depth of sequencing, making lower abundance organisms, as low as 0.1% of a community, tractable for genome reconstruction in the absence of cultivation [2,6,7]. This, in combination with new bioinformatics methods, makes it possible to begin to explore the roles for previously obscure organisms within complex systems such as sediments.

Chloroflexi have been identified from many environments through 16S rRNA gene profiling, including marine and freshwater sediments [8-11]. Despite this, the Chloroflexi remain a relatively understudied bacterial lineage. At present, there are 19 complete genomes available for the Chloroflexi, ranging from the small approximately 1.3 Mb genomes from *Dehalococcoides mccartyi* strains to the giant 13.7 Mb genome from the spore-forming, aerobic *Ktedonobacter racemifer* strain SOSP1-2 [12-16]. The phylum contains a diverse assemblage of organisms with varied metabolic lifestyles, including photoautotrophs like *Chloroflexus aurantiacus*[17], the fermentative *Anaerolinea thermophila* UNI-1 [18], the organohalide respiring organisms in the Dehalococcoidia [12-14], and aerobic thermophiles like *Thermomicrobium*[19]. In sediment systems, only the role of the Dehalococcoidia class is well understood: members of this class rely exclusively on the anaerobic respiration of halogenated hydrocarbons. The broader distribution of this and other metabolic functions across the phylum remain unclear.

The Rifle Integrated Field Research Challenge (IFRC; Rifle, CO, USA) is a uranium-contaminated aquifer with groundwater flow into the Colorado River. Previous research at the site has focused overwhelmingly on the effects of acetate amendment to the groundwater for biostimulation of uranium-respiring microorganisms [20-22]. In contrast, the composition and metabolic potential of the microbial community in regions unaffected by exogenous organic carbon amendment are relatively unknown. Sediment in these regions represents the “background” condition against which acetate amendments and other perturbations have taken place.

Here, we expand the Chloroflexi radiation through metagenomic sequencing of heterogeneous floodplain sediments deposited by the Colorado River. We examine the phylogenetic diversity and predict the metabolic flexibility of the Chloroflexi from this sediment environment, a location that shares similarities with fluvial aquifers worldwide. We curated near-complete genomes from three novel branches of the Chloroflexi, two of which were chosen for their relatively close relationship to the Dehalococcoidia, and conducted an in-depth analysis of their metabolic potential. The genome-based metabolic reconstructions conducted here facilitate determination of previously enigmatic roles in carbon and other geochemical cycles for the Chloroflexi in subsurface environments.

## Methods

### Sequence origin

A sediment core was drilled from well D04 at the Rifle IFRC in July 2007, from within a 6-7 m thick aquifer adjacent to the Colorado River (latitude, 39.52876184; longitude, 107.7720832, altitude 1,619.189 m above sea level; [21]). Sediment samples from 4, 5, and 6 m depths were sampled under anaerobic conditions, stored within gas-impermeable sample bags at -80°C, and kept frozen during transport and prior to DNA extraction. For each depth, 10 independent DNA extractions of 7-14 g of thawed sediment sample were conducted using the PowerMax® Soil DNA Isolation Kits (MoBio Laboratories, Inc., Carlsbad, CA, USA) with the following modifications to the manufacturer’s instructions. Sediment was vortexed at maximum speed for an additional 3 min in the SDS reagent, and then incubated for 30 min at 60°C in place of extended bead beating. The 10 replicate DNA samples were concentrated using a sodium acetate/ethanol/glycogen precipitation and then pooled for sequencing, generating one pooled DNA sample from approximately 100 g of sediment per depth.

### Assembly metrics, gene calling, and binning

Four lanes of Illumina HiSeq paired-end sequencing were conducted by the Joint Genome Institute. The 4 m sample sequence comprised 360,739,614 reads, the 5 m sample 497,853,726 reads, and the 6 m sample 140,430,174 reads. The read length was 150 bp. Reads were preprocessed using Sickle (https://github.com/najoshi/sickle) using default settings. Only paired end reads were used in the assemblies. Most of our analyses relied upon IDBA_UD assemblies using default parameters [23] of sequence data for the 4 m sample, the 5 m sample, and the 6 m data separately. One barcoded lane of Illumina HiSeq paired-end sequence containing all three of the depth samples was co-assembled using the IDBA_UD assembler [23] using two different parameter settings: mink 40, maxk 100 (Combo1 assembly) and mink 40, maxk 100, min_count 2 (Combo3 assembly). Combo1 was only used for binning; Combo3 was used during genome curation.

Emergent self-organizing map (ESOM) clustering based on tetranucleotide frequencies of scaffolds produced in the Combo1 assembly was used to identify segregated clusters of scaffolds corresponding to individual genomes [24]. Chloroflexi scaffolds were identified using taxonomic affiliation of genes predicted with Prodigal (meta-Prodigal option; [25]) based on best blast match, where 40% of genes were required to have a match to Chloroflexi sequences in order for a scaffold to be included. GC content, abundance profile in the 4, 5, and 6 m depth metagenomes, and the taxonomic affiliations of the genes encoded on scaffolds were used to curate a consistent set of scaffolds with a high proportion of Chloroflexi-affiliated predicted genes.

Three Chloroflexi genomes were selected for curation and characterization based on their positions as relatively phylogenetically novel organisms within the Chloroflexi, the presence of a clearly defined ‘genome’ bin within the ESOM analysis, and the taxonomic predictions for the genes within the genomes. For each genome bin, the paired reads from all depth samples that mapped to the genomes’ scaffolds were reassembled. RBG-2 reads were assembled using IDBA_UD under default parameters [23] (Rifle BackGround organism # (RBG)). The RBG-9 and RBG-1351 reads were assembled using Velvet [26]. For each genome, mini assemblies using all reads mapping to the ends of scaffolds were conducted until no further connections between scaffolds could be made. Genome completion was examined with a suite of 76 genes selected from a set of single copy phylogenetic marker genes that show no evidence of lateral gene transfer [27,28].

A functional prediction was conducted on open reading frames on scaffolds of interest. This involved amino-acid similarity searches against UniRef90 [29] and KEGG [30,31]. Additionally, UniRef90 and KEGG were searched back against the translated sequences to identify reciprocal best-blast matches. Reciprocal best blast matches were filtered with a minimum 300 bit score. One-way blast matches were filtered with a minimum 60 bit score. The translated sequences were also submitted to motif analysis using InterproScan [32]. tRNA sequences were predicted using tRNAscan-SE [33]. Finally, the annotation summaries were ranked: reciprocal best-blast matches were ranked the highest, followed by one-way matches, followed by InterproScan matches, followed by just a gene prediction (annotated as hypothetical proteins).

### Gene-specific phylogenies

For specific functional genes of interest, reference datasets were generated from sequences mined from NCBI databases. In all cases, the nearest homolog within the Chloroflexi was determined and included in the reference set; absence of Chloroflexi within these datasets indicates there were no identifiable homologs. Alignments were generated using MUSCLE v. 3.8.31 [34,35], curated manually, and phylogenies conducted using PhyML [36] with 100 bootstrap resamplings.

### Concatenated ribosomal protein phylogeny

Existing reference datasets for the 16 ribosomal proteins chosen as single-copy phylogenetic marker genes (RpL2, 3, 4, 5, 6, 14, 15, 16, 18, 22, and 24, and RpS3, 8, 10, 17, and 19) [27,28] were augmented with sequences mined from recently sequenced genomes from the Chloroflexi, Nitrospirae, and TM7 phyla, among others, from the NCBI and JGI IMG databases. Each individual gene set was aligned using MUSCLE version 3.8.31 [34,35] and then manually curated to remove end gaps and ambiguously aligned regions. Model selection for evolutionary analysis was determined using ProtTest3 [37,38] for each single gene alignment. The curated alignments were concatenated to form a 16-gene, 930 taxa, 2,456-position alignment. A maximum likelihood phylogeny for the concatenated alignment was conducted using PhyML under the LG + α + γ model of evolution and with 100 bootstrap replicates [36].

### Ribosomal protein S3 phylogeny

RpS3 sequences were mined from the JGI IMG-M site from all available metagenome sequences, excluding human microbiome samples, using the gene name search tool. In cases where multiple assemblies or samples were available for the same environmental site, a subset of representative metagenome assemblies was selected. A total of 7,707 RpS3 sequences were identified. After removing protein sequences shorter than 200 aa, 1,152 partial and full length RpS3 sequences were searched against the NCBI nr protein database using BLASTp [39]. The sequences were aligned with the RpS3 reference set as described above, and the Chloroflexi-affiliated sequences identified using a combination of a Neighbor-Joining Jukes-Cantor tree and MEGAN [40] on the BLASTp data. The final dataset of 794 sequences was aligned and masked, and the best fitting evolutionary model determined as described above. A maximum likelihood phylogeny was conducted using PhyML under the LG + α + γ model of evolution, with 100 bootstrap replicates [36]. For coverage calculations, Bowtie2 [41] was used to map all reads from each depth (as singletons) to a dataset comprising all of the RBG scaffolds containing the *rpS3* genes. Coverage levels were normalized across the three datasets for total number of reads, and the relative ratio and abundances determined.

## Results and discussion

### An expanded view of the Chloroflexi phylum

Three 1.5 kg sediment samples, consisting of unconsolidated sands, silts, clays, and gravels deposited by the Colorado River, and containing identifiable woody debris (for example, twigs, bark, roots) within the fine-grained, silt-dominated matrix were collected 4, 5, and 6 m below the ground surface. Assemblages of fine-grained sediments and refractory organic matter are characteristic features of fluvial overbank deposits, a common aquifer architecture, with subsequent sediment deposition leading to burial of organic matter of variable carbon content and quality. These samples allowed for examination of the microbial community present in the native sediment prior to acetate amendments, a more representative state for the Rifle sediment and for aquifers in general.

**Figure 1:**
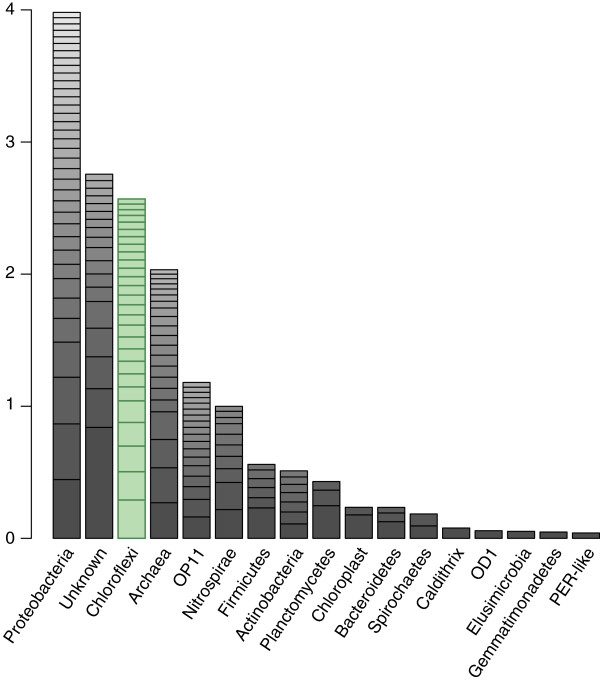
**Rank abundance curve of the 161 most abundant organisms from the 5 m metagenome assembly for which at least eight of the 16 selected ribosomal proteins were recovered.** Bars correspond to summed abundances for major taxonomic divisions (domains and phyla), with individual organism abundances denoted by stacked boxes. Taxonomic affiliation for each organism was determined based on placement on the concatenated ribosomal protein tree. Abundance metrics were calculated utilizing total dataset size, coverage of ribosomal protein-containing scaffolds, and predicted genome sizes (3 Mbp in general, 1.5 Mbp for OD-1 and OP-11). Ribosomal proteins utilized were RpL2, 3, 4, 5, 6, 14, 15, 16, 18, 22, 24 and RpS3, 8, 10, 17, 19.

Assembly of metagenomic data from the 4 , 5 , and 6 m depth intervals yielded 415, 740, and 82 Mbp of contiguous sequence, respectively, in scaffolds >5 kb in length. From a rank abundance analysis based on the ribosomal protein S3 (rpS3), Chloroflexi represented a significant fraction of the most abundant organisms in the aquifer sediment: 25 of 160 organisms (16%) in the 4 m sample; 34 of 238 (14%) in the 5 m sample (Figure 1); and 3 of 35 (9%) in the 6 m sample. Across all three datasets, the Chloroflexi (14%) were the second-best represented bacterial phylum after the Proteobacteria (23%). No one organism represented greater than 1% of the total community.

**Figure 2:**
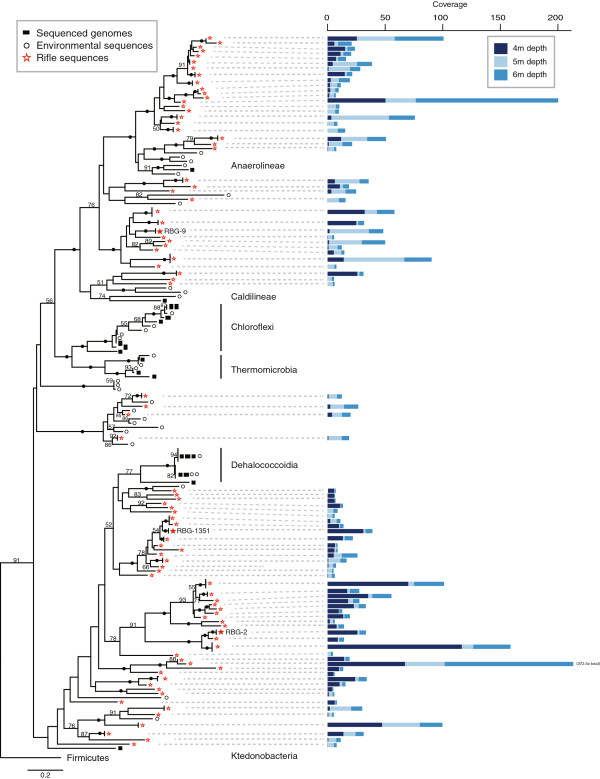
**Chloroflexi diversity and abundance from the Rifle sediment metagenomes.** Left: Maximum likelihood phylogeny of the Chloroflexi phylum based on an RpS3 protein alignment. Bootstrap values >50 are displayed, with bootstraps >95% denoted by a black circle. Rifle-derived RpS3 sequences are marked with red stars, complete genomes with filled squares, and environmental sequences mined from NCBI and IMG/M with empty circles. See Methods for alignment and phylogeny details, and Additional file 1: Figure S1 in the supplemental online material for full sequence names. Right: Stacked bar charts of normalized coverage values for each *rpS3*-containing scaffold in each depth’s metagenome.

On scaffolds >5 kb in length, 86 full-length unique Chloroflexi *rpS3* (single copy genes) were identified, indicating the presence of at least this many distinct genotypes in the sediment. There were 139 Chloroflexi sequences included in the RpS3 protein phylogenetic analysis; 19 from sequenced genomes of known organisms, 34 from environmental samples mined from IMG/M, and the 86 sequences from Rifle sediment metagenomes. The Rifle sequences represent 62% of the Chloroflexi for which an *rpS3* gene was identified and clearly expand the diversity of the phylum, including new genera, family, and class-level clades not currently represented by sequenced genomes (Figure 2, Additional file 1: Figure S1). Detection of all lineages at all three depths was possible using read-mapping. This approach, which only requires that the genomic fragment carrying the *rpS3* be assembled in at least one of the samples, allows detection of an organism present at an abundance level of 0.02%. The Chloroflexi showed broad differences in abundance across the depth of the aquifer, with organisms more closely related to the Anaerolinea more abundant in the deeper 5 and 6 m samples, and organisms related to the Dehalococcoidia and uncultured classes most abundant in the shallower 4 m sample (Figure 2).

### A protein gene method for taxonomic profiling of metagenomes

The 16S rRNA gene is the benchmark for placement of novel organisms within the microbial tree of life [42,43]. In metagenomic studies, 16S rRNA genes typically do not assemble well [44], or, if assembled in a separate analysis (for example, EMIRGE [45]), are difficult to connect to their respective genomes. A protein tree built from a single gene (for example, RpS3) can examine microbial diversity, but does not provide sufficient information for resolution of deep divergences. To address this, the Chloroflexi-affiliated *rpS3* genes served as anchors to identify metagenome scaffolds containing a conserved, syntenic block of ribosomal proteins. Eighty of the 86 RpS3 lineages were represented by genes on scaffolds encoding >50% of the 16 ribosomal protein genes of interest, and thus were included in a concatenated protein alignment for phylogenetic analysis (Additional file 1: Figure S2). The 16-ribosomal-protein concatenated alignment yielded a phylogenetic tree with resolution commensurate with that of a 16S rRNA tree (Additional file 1: Figures S2 and S3). The Chloroflexi are strongly supported as a monophyletic clade, including support for the recent reassignment of the genus *Ktedonobacter* to this phylum [46]. Additionally, the ribosomal proteins provide a stable marker for assessment of genomic sampling within the metagenome based on scaffold coverage (Figure 2). The scaffolds encoding *rpS3* genes ranged from 5,000 to 162,967 bp in length (average 20,806 bp, standard deviation 24,847), and most had coverage of 7-10× or higher.

The use of stable single-copy protein genes in lieu of 16S rRNA genes is not novel [28,47,48]. However, this syntenic 16-gene block has advantages over other combined protein-gene analyses: it affords relatively deep resolution of diverse bacterial and archaeal lineages while simultaneously removing any requirement for binning, making it a powerful option for taxonomic assessment of metagenomes.

### Genome sequences of three novel Chloroflexi

Three draft genomes were curated from the sediment assemblies, and are named RBG-2, RBG-9, and RBG-1351. RBG-2 and RBG-1351 were most abundant in the 4 m depth sample, while RBG-9 was most abundant in the 5 m sample (Table 1). Single copy marker gene analysis identified 74, 69, and 75 out of 76 marker genes in the draft RBG-2, RBG-1351, and RBG-9 genomes, respectively (see Additional file 1: Tables S1-3 for lists of markers). We estimate that all three genomes are >90% complete.

**Table 1:** Statistics for the three RBG Chloroflexi genomes

**Scaffold length distribution**	**RBG-2**	**RBG-1351**	**RBG-9**
500-1,000	190	0	1
1,000-5,000	141	0	4
5,000-10,000	2	3	5
10,000-20,000	0	14	10
20,000-50,000	5	20	5
50,000-100,000	4	8	11
100,000-200,000	6	1	12
200,000+	2	0	4
General information			
Abundance ratio (4 m:5 m:6 m)	78:6:16	80:5:15	4:70:26
Scaffolds (*n*)	350	46	52
Total bp (*n*)	2,314,541	1,510,752	3,831,943
Avg. seq. length (bp)	6,613	32,842	73,691
N50 (bp)	156,509	42,166	148,655
Maximum scaffold size (bp)	304,207	159,726	354,139
Scaffolds >2500 (>10,000)			
Scaffolds (*n*)	37 (17)	46	48 (42)
Total bp (*n*)	2,008,616 (1,936,016)	1,510,752	3,825,973 (3,785,678)
Avg. seq. length	54,286 (113,883)	32,842	79,707 (90,135)
N50	163,466 (same)	42,166	148,655 (same)
Genome characteristics			
G + C content (%)	1,022,478 (50.8)	749,453 (49.6)	2,344,523 (61.2)
Genes	2,134	1,635	3,646
Protein coding genes	2,068	1,603	3,596
Genes with functional prediction	1,409	1,069	1,535
Signal P^a^	47 (2.3%)	39 (2.4%)	144 (3.9%)
Trans-membrane proteins^b^	356 (17.2%)	302 (18.8%)	918 (25.1%)
Tat signal proteins^c^	4 (0.2%) (179 (8.7%))	1 (0.06%) (25 (1.5%))	27 (0.7%) (143 (3.9%))
rRNA genes (5S/16S/23S)	0/1/1	0/0/0	0/1/1
tRNA genes (*n*)	64	32	48

^a^Data obtained using SignalP 3.0 [49,50].

^b^Data obtained using TMHMM Server v. 2.0.

^c^Data obtained using TatP 1.0, numbers in brackets represent potential Tat proteins with no Tat motif [51].

### RBG-2

RBG-2 belongs to the GIF9 Class [52] based on 16S rRNA gene phylogeny (Additional file 1: Figure S3). The GIF9 are a clade of uncultured Chloroflexi that have been identified in a myriad of environments, including marine sediments and methane seeps, freshwater sediments, hypersaline environments, bioreactors, and oil sands tailings [53-58]. Several studies have postulated a link between GIF9 Chloroflexi and organic-rich sediments, typically marine and/or methane-impacted [59-61], but currently their role within these communities is unknown. There is no available sequenced genome for this group; the most closely-related genomes are from the class Dehalococcoidia, including *Dehalogenimonas lykanthroporepellens*[62] and various *Dehalococcoides mccartyi* strains’ genomes [12-14]. RBG-2 shares 84% 16S rRNA gene sequence identity with *Dehalococcoides* spp. and *Dehalogenimonas lykanthroporepellens* BL-DC-9, the closest available sequenced genomes, and 96% sequence identity with the nearest database 16S rRNA gene sequence, a GIF9 organism from a limestone sinkhole in northeastern Mexico (Additional file 1: Figure S3).

**Figure 3:**
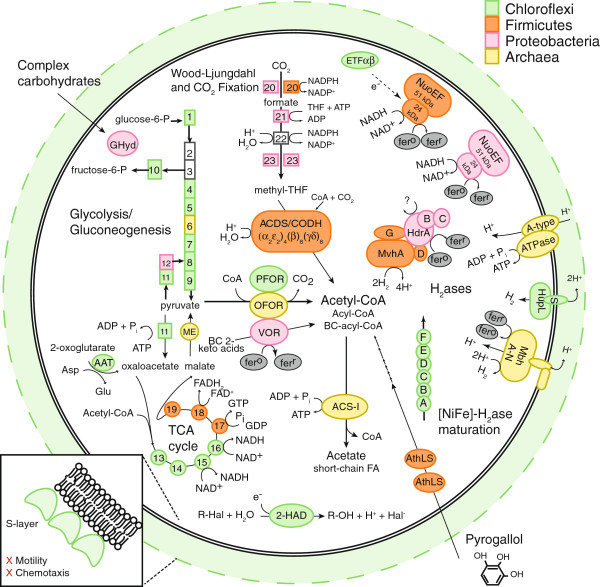
**Predicted energy metabolism for RBG-2.** Proteins and complexes are colored based on the taxonomic affiliation of the best blast match from the NCBI nr database (white = no clear Phylum affiliation). Abbreviations: 1 - glucose phosphate isomerase, 2 - phosphofructokinase, 3 - fructose bisphosphate aldolase, 4 - triose phosphate isomerase, 5 - glyceraldehyde-3-phosphate dehydrogenase, 6 - phosphoglycerate kinase, 7–2,3-bisphosphoglycerate-dependent phosphoglycerate mutase, 8 - enolase, 9 - pyruvate kinase, 10 - fructose-1,6-bisphosphatase I, 11 - pyruvate carboxylase, 12 - PEP carboxykinase, 13 - RE-citrate synthase, 14 - aconitate hydratase 1, 15 - isocitrate dehydrogenase, 16 - 2-oxoglutarate:ferredoxin oxidoreductase, 17 - succinyl-CoA synthetase, 18 - succinate dehydrogenase, 19 - fumarate hydratase, 20 - formate dehydrogenase, 21 - formyltetrahydrofolate synthetase [formate-tetrahydrofolate ligase], 22 - bifunctional methenyltetrahydrofolate cyclohydrolase/methelynetetrahydrofolate dehydrogenase, 23 - methylenetetrahydrofolate reductase, 2-HAD - 2-haloacid dehalogenase, AAT - aspartate amino transferase, ACDS-I - acetyl-CoA synthetase (ADP-dependent), ACDS/CODH - acetyl-CoA decarbonylase/synthase/carbon monoxide dehydrogenase complex, AthLS - pyrogallol hydroxytransferase large and small subunits, ETF - electron transporting flavoprotein, GHyd - glycoside hydrolase, Hdr - heterodisulfide reductase, Hup - Uptake hydrogenase, Mbh - membrane-bound hydrogenase, ME - malic enzyme, Mvh - F420 non-reducing hydrogenase, OFOR - oxo-acid:ferredoxin oxidoreductase, PFOR - pyruvate:ferredoxin oxidoreductase, THF - tetrahydrofolate, VOR - 2-ketoisovalerate:ferredoxin oxidoreductase.

The RBG-2 genome encodes more metabolic potential than the genomes of the Dehalococcoidia [13,63], for which hydrogen and halogenated compounds are the sole electron donor/acceptor pair utilized. RBG-2 is likely reliant on a fermentative metabolism, lacking a standard electron transport chain including cytochromes and all membrane-bound Complex I subunits (see Additional file 2). The genome contains two NuoEF operons, the so-called ‘FP fragment’ of Complex I, responsible for NADH dehydrogenase activity [64]. The function of the FP fragment is unclear, but it may serve to regenerate NADH. In terms of a TCA cycle, the RBG-2 genome contains a predicted RE-citrate synthase, as seen in the *Dehalococcoides*[65], and lacks a bacterial malate dehydrogenase for the conversion of malate to oxaloacetate. Instead, malate is likely converted to pyruvate and CO_2_ by an Archaeal-type decarboxylating malate dehydrogenase (malic enzyme). In the absence of PDH, pyruvate may subsequently be converted to oxaloacetate by pyruvate carboxylase [66] or to acetyl-CoA and CO_2_ by a pyruvate:ferredoxin oxidoreductase (PFOR, subunits αβδγ) (Figure 3).

Based on the lack of annotated proteins implicated in respiratory metabolism, we conclude RBG-2 is an obligate fermenter reliant on sugars and plant polymers for energy generation. To this end, the RBG-2 genome contains a complete glycolysis pathway, as well as gluconeogenesis from pyruvate to fructose-6-phosphate (Figure 3). The genome also encodes two pyrogallol-phloroglucinol transhydroxylase complexes that are structurally similar to the enzyme from *Pelobacter acidigallici*, an anaerobic Deltaproteobacterium that ferments pyrogallol to phloroglucinol and subsequently to three moles of acetate (plus CO_2_) [67-69]. Pyrogallol is a component of plant polymers and may serve as an important energy source for RBG-2 given its presence in a shallow aquifer system containing buried organic material.

Beyond the fermentation of sugars, RBG-2 encodes the mechanism for degrading fatty acids and organic acids to generate acetyl-CoA. Even- and odd-chained saturated fatty acids are predicted be degraded to acetyl-CoA via a complete beta-oxidation pathway (see Additional file 2). Organic acids are predicted to be decarboxylated via a multitude of ferredoxin-oxidoreductases present on the RBG-2 genome, including PFOR, two 2-oxoacid:acceptor oxidoreductases (OFOR), 2-ketoisovalerate:ferredoxin oxidoreductase (VOR), five aldehyde ferredoxin oxidoreductases (AOR), and two archaeal-affiliated2-oxoglutarate:ferredoxin oxidoreductases (KGOR). These enzymes ultimately produce acetyl-CoA and derivatives, CO_2_, and reduced ferredoxin [70-73].

RBG-2 may also produce acetyl-CoA by reducing CO_2_ via a complete Wood-Ljungdahl pathway (Figure 3). Key enzymes include two selenocysteine-containing formate dehydrogenases and an acetyl-CoA decarbonylase synthase/CO dehydrogenase (ACDS/CODH) complex [74]. It is possible that this pathway functions heterotrophically or autotrophically in RBG-2. Under heterotrophic conditions, the pathway reduces intracellular CO_2_ pools and oxidizes reduced ferredoxin and NADH generated by glycolysis or decarboxylation reactions (mentioned above). RBG-2 may be capable of chemolithoautotrophic growth, reducing external CO_2_ pools using reductant from Ni,Fe hydrogenases (for example, Hup, Mvh), yielding acetyl-CoA [74] (see Additional file 2).

We propose that acetate is the primary fermentation end product of RBG-2 metabolism, as genes to produce ethanol, lactate, formate, or butyrate were absent. Acetyl-CoA and derivatives produced from the breakdown of complex polymers, sugars, fatty acids, organic acids, and CO_2_ may be funneled to an ADP-dependent acetyl-CoA synthetase I (ACS-I) to produce acetate and yield ATP by substrate level phosphorylation. Acetyl-CoA, acyl-CoA, and branched-chain acyl-CoA, formed by PFOR, OFOR, and VOR respectively, were all shown to be substrates for ACS-I and ATP formation in *Pyrococcus furiosus*[75], and may be in RBG-2 as well. This enzyme is more commonly found in Archaea [47], but has recently been annotated in metagenome-derived representatives from a newly defined phylum called RBG-1 [76] and from members of the OD1 candidate phylum [2]. In order to supplement ATP from fermentation, RBG-2 may use a group IV multi-subunit membrane-bound Ni,Fe hydrogenase (Mbh) to generate a proton motive force, which can fuel oxidative ATP formation by a predicted ATP synthase of Crenarchaeotal origin (Figure 3, Additional file 2, Additional file 1: Figures S4-6).

The current study provides the first genome-based prediction for the roles of GIF9 Chloroflexi in sediment. From the evidence outlined above, we hypothesize that RBG-2 is a fermenter, specifically a homoacetogen. This is the first documentation of this metabolism in the Chloroflexi, but could explain the wide abundance of GIF9 in organic-rich anoxic sediments. Consistent with a homoacetogenic lifestyle, the RBG-2 genome encodes diverse mechanisms for generation of acetyl-CoA, including fermentation of glucose and pyrogallol, beta-oxidation of fatty acids, as well as biosynthesis from CO_2_. The presence of a pyrogallol degradation pathway, in conjunction with a glycoside hydrolase, indicates RBG-2 may be saprophytic within the sediment environment, harvesting energy from dead plant remains.

### RBG-1351

RBG-1351 represents the current nearest genomic neighbor to the *Dehalococcoidia,* based on the concatenated ribosomal protein tree. In the absence of a genome-associated 16S rRNA gene it is not currently possible to assign RBG-1351 to a described Chloroflexi class, although we hypothesize that it falls within either the GIF3 or vadinBA26 class based on an EMIRGE analysis (Additional file 1: Figure S3).

Like RBG-2, RBG-1351 is predicted to be an obligate fermenter lacking electron transport chain components and a complete oxidative or reductive TCA cycle. In addition to the NuoEF genes identified in RBG-2, the RBG-1351 genome contains homologs for the L, M, and N membrane anchor subunits of Complex I. The absence of the connecting subunits (D,C,I,B) make it unlikely the membrane-associated NuoLMN subunits interact with the NuoEF proteins. The fragmented TCA cycle includes an RE-citrate synthase like that of RBG-2, but is missing aconitate hydratase, succinyl-CoA synthetase, and a reverse citrate lyase.

Unlike RBG-2, we could not recover a complete canonical glycolysis pathway in the RBG-1351 genome, as genes for fructose bisphosphate aldolase and pyruvate kinase were not identified. The conversion of fructose-6-phosphate to glyceraldehyde-3-phosphate may instead be catalyzed by transaldolase, allowing glycolysis to proceed to phosphoenol-pyruvate (PEP), but a gene for the conversion of PEP to pyruvate was not identified, suggesting either pyruvate kinase is missing or poorly annotated on the >90% complete RBG-1351 draft genome, or perhaps RBG-1351 uses a different mechanism for producing pyruvate via glycolysis. The RBG-1351 genome does encode PFOR, which, given the critical gluconeogenesis enzyme pyruvate carboxylase was not identified, indicates pyruvate is likely converted to acetyl-CoA (see Additional file 2).

Despite their phylogenetic distance (Figure 2), RBG-2 and RBG-1351 share several mechanisms for generating acetyl-CoA and both are likely acetogens. Similar features shared between the genomes include a complete Wood-Ljungdahl pathway, beta-oxidation of saturated fatty acids, and the presence of two operons for pyrogallol-phloroglucinol transhydroxylase complexes (see Additional file 2). Relative to RBG-2, RBG-1351’s genome contains fewer ferredoxin:oxidoreductases, encoding one IOR and six AORs for producing acetyl-CoA from organic acids. Ultimately, like RBG-2, RBG-1351 is predicted to convert acetyl-CoA to acetate using ACS-I with the concomitant formation of ATP (Additional file 1: Figure S7).

Despite the similarity in broad functionality between the two genomes, RBG-1351 contains features not identified in the RBG-2 genome. Unlike the Hup, Mvh, and Mbh hydrogenases in RBG-2, we identified a group I membrane-bound Ni,Fe hydrogenase (HydABC) encoded in the RBG-1351 genome. Group I hydrogenases function as H_2_ uptake hydrogenases in respiratory hydrogen oxidation, often in concert with a fumarate reductase, quinone pool, or nitrate reductase [77]. The RBG-1351 genome does not encode any of these; the electron partner and hence the role for this hydrogenase is unclear in this proposed obligatory fermentative organism. Group I hydrogenases are commonly identified in Chloroflexi genomes, including those of the Dehalococcoidia, who also lack a canonical electron partner for this enzyme. Unlike RBG-2, the ATP synthase encoded on the genome is a F_0_F_1_ bacterial type. The RBG-1351 genome encodes an additional pathway for generating acetyl-CoA not found in RBG-2 genome: an acetone carboxylase operon (*hyuABC*) whose enzyme products may detoxify acetone to acetoacetate [78]. Acetoacetate may subsequently be converted to acetyl-CoA via 3-oxoacid CoA transferase and acetyl-CoA C-acetyltransferases. The genome encodes three acetylene dehydratases homologous to an enzyme in *Pelobacter acetylenicus*, though prediction of putative substrates for these enzymes is not possible (see Additional file 2). In addition to acetate, RBG-1351 may also produce propionate via succinyl-CoA (see Additional file 2).

The highly similar metabolisms predicted for RBG-1351 and RBG-2, members of different Chloroflexi classes, indicates the predicted anaerobic acetogenic lifestyle may be relatively widespread within the phylum. The multiple acetyl-CoA-generating pathways within these genomes may be optimal under shifting environmental conditions, for example as seasonal changes alter the water table and nutrient deposition in the sediment. These two organisms were at highest abundance in the shallowest, 4 m sample, and are predicted to be active in degrading plant-derived organic matter. The acetate formed by these organisms is likely excreted, subsequently acting as an electron donor and/or carbon source for other organisms in the sediment. Acetate amendment to the Rifle subsurface results in growth of uranium- and iron-respiring organisms; RBG-2 and RBG-1351’s activities likely support the presence of these respirers in the sediment in the absence of anthropogenic acetate amendment, albeit at much lower abundances.

### RBG-9

RBG-9 is placed within the Chloroflexi class Anaerolinea, members of which have been identified from diverse environments, including arctic permafrost, marine, and freshwater sediments, and anaerobic sludge bioreactors [5,8,11,79]. The type organism for the class, *Anaerolinea thermophila* UNI-1, was isolated from a sludge reactor, and is postulated to function in granule formation as well as contribute to problematic bulking within reactors [18,79]. *A. thermophila* UNI-1 is anaerobic, and grows chemo-organotrophically on amino acids and a variety of carbohydrates [80]. The RBG-9 16S rRNA gene shares 87% sequence identity with *A. thermophila* UNI-1, and 95% sequence identity with the nearest database 16S rRNA gene, from an uncultured bacterium identified in the saturated zone of the Hanford nuclear reactor site in Washington State, USA [81].

The predicted respiratory metabolism from RBG-9 genome analysis presents a substantial contrast to the obligate fermentation predicted from the genomes of RBG-2 and RBG-1351. Notably absent in the RBG-9 genome are key functional genes of the Wood-Ljungdahl pathway, PFOR, and hydrogenases. In RBG-9, ATP synthesis is predicted to proceed primarily via oxidative phosphorylation, using an aerobic electron transport chain containing a pyruvate dehydrogenase complex, a 14 subunit NADH dehydrogenase Complex I, a succinate dehydrogenase/fumarate reductase complex II, two PetAB complexes (cytochrome *b*-Rieske type complexes, for example, quinol:electron acceptor oxidoreductase), a *caa*_*3*_-type cytochrome *c* oxidase, and an F_0_F_1_-ATP synthase. The F_1_ δ subunit is missing, and the ϵ subunit disrupted by scaffolding. Also present is an alternative complex III (ACIII) which performs the same function as the bc_1_ complex and clusters on a DMSO reductase tree with proteins from iron respiring Deltaproteobacteria ([82], Additional file 1: Figure S8, Additional file 2).

Notable in the RBG-9 genome is the diversity of cytochromes: beyond the cytochrome *c* oxidase and cytochrome *b*-rieske complex, the genome encodes several predicted multiheme *c*-type cytochromes, including three monoheme cytochromes, two dihemic cytochromes, a decaheme, and a 24-heme cytochrome. Also present are two genes encoding predicted octaheme *c*-type complexes in operons with subunits from the DMSO reductase family. In addition to the ACIII, RBG-9 possesses four other genes encoding molybdopterin oxidoreductases: one related to polysulfide and thiosulfate reductases [83], while the other three are most similar to formate dehydrogenases (Additional file 1: Figure S8). Such predictions are provisional and require further experimental testing, however, these membrane-bound complexes may function in respiration under anoxic conditions.

The RBG-9 genome contains a complete suite of genes that code for enzymes in glycolysis, gluconeogenesis, and the lower half of the pentose-phosphate pathway. Within a complete TCA cycle, the genome contains a predicted classic citrate synthase, unlike the RE-citrate synthases found in the other two genomes. Genes for sugar metabolism are extensive, including those encoding enzymes involved in the utilization of fructose, galactose, maltose, sorbitol, starch, sucrose, trehalose, xylose, and xylulose. The genome contains two predicted endoglucanases (cellulases) as well as a beta-glucosidase for complex carbohydrate degradation to sugar monomers. RBG-9 does not encode a pyrogallol-phloroglucinol hydroxytransferase, though a beta subunit is present at the end of one scaffold, hinting that this function may be present on the complete genome. The RBG-9 genome contains the genes required for beta-oxidation of odd- and even-chain saturated fatty acids (KEGG pathway map00071). Similar to the other two genomes examined here, genes for enzymes active in metabolism of unsaturated fatty acids were not identified. The genome contains genes for a single indoylpyruvate ferredoxin:oxidoreductase (IOR), two AORs, two KGORs, and 35 uncharacterized oxidoreductases. RBG-9 encodes an ADP-dependent ACS-I similar to RBG-2 and RBG-1351, for acetate fermentation with substrate-level phosphorylation of ATP in anaerobic conditions (Additional file 1: Figure S7). In aerobic conditions, the acetyl-CoA is likely funneled to the complete TCA cycle for aerobic oxidation.

RBG-9 may be capable of respiration and fermentation, with a complete pathway for sugar fermentation to propionate encoded on the genome, including a methylmalonyl-CoA decarboxylase coupled to Na^+^ extrusion and proton motive force generation. RBG-9 may also utilize amino acids (specifically, lysine, glutamine, 2-oxoglutarate, alanine, and aspartate) in several fermentative pathways (see Additional file 2). Aside from a diverse suite of peptide and amino acid transporters, the genome encodes genes for many predicted peptidases, proteases and protein kinases (49, 19, and 20, respectively). These may exist in part to compensate for absent amino acid synthesis pathways (see Additional file 2), as well as to provide fermentation substrates as an alternative to sugar catabolism.

Beyond sugar and amino acid degradation, the RBG-9 genome contains several genes associated with detoxification, degradation of contaminants, and heavy metal redox, including an NAD(P)H-dependent mercuric reductase and a copper nitrite reductase. The RBG-9 genome contains homologs to *atzA* and *atzB*, N-ethylammeline chlorohydrolase, and hydroxyatrazine ethylaminohydrolase, the first two steps in atrazine degradation [84]. Homologs to *atzC* or the downstream cyanuric acid degradation pathway were not identified. These enzymes, along with an arsenic resistance pathway (see Additional file 2), may provide RBG-9 with protection from environmental toxins present in the contaminated aquifer or synthesized by other microorganisms.

Uncultured *Anaerolinea* sp. have been shown to scavenge organic compounds from decaying cell remains [85], a role that RBG-9 may also fulfill through import and catabolism of cellulose and cellulosic derivatives, sugars, starch, peptides, and amino acids. RBG-9 is predicted to utilize a variety of sugars, coupled to reduction of oxygen for energy generation. RBG-9 was most abundant in the 5 m depth sample, well below the water table in a region of the aquifer where anaerobic conditions are expected. Sugar and amino acid fermentation pathways may provide an anaerobic adaptation for RBG-9’s survival in the shifting aerobic conditions of the sediment.

### Membrane architecture and motility

The Chloroflexi were classically defined as Gram-negative staining, single membrane organisms [86], often with unusual membrane lipids [19,87] and an absence of lipopolysaccharide. Several recently described Chloroflexi, including species from *Ktedonobacter*, *Nitrolancetus*, *Sphaerobacter*, and *Thermobaculum* stain Gram-positive, and contain a thin layer of peptidoglycan within their cell walls [16,88-90]. Most described Chloroflexi are non-motile, with the exception of gliding motility seen in the class Chloroflexi (for example, *Chloroflexus, Herpetosiphon*) [17,91].

**Figure 4:**
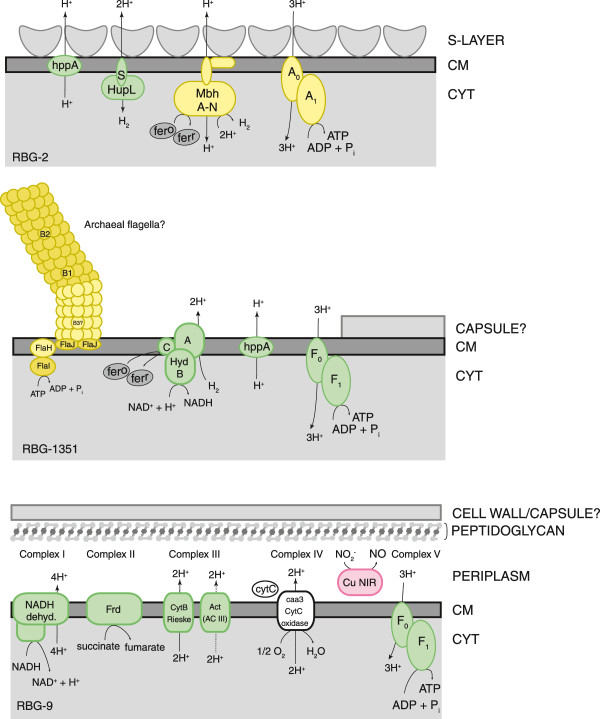
**Predicted membrane structure and proton motive force mechanisms from the three reconstructed Chloroflexi genomes.** All three Chloroflexi are predicted to be Gram-negative, single membrane bound cells. RBG-9 is the only genome containing a predicted peptidoglycan pathway. Abbreviations: A_0_A_1_ - archaeal-type ATP synthase, Act - alternative complex III (ActABCDE), CM - cell membrane, Cu NIR - copper nitrite reductase, CYT - cytoplasm, CytC - cytochrome c, F_1_F_0_ - bacterial-type ATP synthase, Frd - succinate dehydrogenase/fumarate reductase (subunits ABC), hppA - membrane-bound proton-translocating pyrophosphatase, Hup - uptake hydrogenase, Hyd - Ni, Fe-hydrogenase, Mbh - membrane bound hydrogenase.

None of the three draft genomes contain pathways for lipopolysaccharide biosynthesis or spore formation. In keeping with the classical Chloroflexi model, RBG-2 and RBG-1351 do not encode the peptidoglycan synthesis pathway. RBG-2 encodes genes associated with formation of S-layer glycoproteins. The RBG-1351 genome contains evidence for a cell wall, in the form of two putative cell wall repeat proteins, while no genes encoding for S-layer glycoproteins were identified. In contrast, RBG-9 encodes a complete peptidoglycan synthesis pathway (Figure 4). From this, and the presence of 15 distinct inner membrane translocation/transport proteins, RBG-9 is hypothesized to construct a layer of peptidoglycan, a multi-layered cell envelope structure seen in several other Chloroflexi [16,19,88,90]. The RBG-9 genome also contains 48 predicted glycosyl transferases as well as 15 other genes associated with cell wall and/or capsule formation. A predicted indigoidine synthase suggests the possibility that RBG-9 is pigmented. The presence of two *lon* genes, associated with filament formation, as well as an operon of *mreBCD* (rod-shape determining proteins) with *minCDE* (septum site determining proteins) suggests RBG-9 may form filaments similar to *Anaerolinea* and *Caldilinea* organisms [18].

RBG-2 and RBG-9 are predicted to be non-motile. One of the most striking features on the RBG-1351 genome is the presence of an operon encoding the majority of an archaeal flagellar apparatus [92]. This includes the motor proteins (FlaHIJ), assembly proteins (FlaF, FlaG), and three flagellins (FlaB1, FlaB2, and an unclassifiable FlaB). Genes encoding the minor flagellin (FlaA) and several accessory proteins (FlaCDE and FlaK) are not present. The absence of a gene for FlaK, the preflagellin peptidase, calls into question the ability of RBG-1351 to generate a flagellum [92]. Paired-end read mapping confirmed this operon is situated on a scaffold that is otherwise clearly Chloroflexi in origin, and which binned robustly with the RBG-1351 scaffolds. Flagellar apparatus operons were identified in the *Thermomicrobium roseum* and *Sphaerobacter thermophilus* genomes, two non-motile Chloroflexi [19,89]. These flagellar genes are bacterial in origin (*flg* genes), unlike the system described here (*fla* genes). The archaeal-type flagella, if utilized, would theoretically be compatible with the RBG-1351 single membrane architecture.

Further details on the three Chloroflexi genomes, including predicted amino acid and cofactor biosynthesis, transporters, transcriptional regulation, phage and mobile element signatures, oxygen tolerance and free radical scavenging mechanisms, and taxonomic affiliations for metabolic genes of interest can be found in the supplemental electronic materials.

### Sediment chloroflexi metabolic potential

A total of 3,571, 2,743, and 1,078 Chloroflexi scaffolds were identified from the 4, 5, and 6 m assemblies, not including genomic information from the curated genomes discussed above. Based on the average number of genes from a suite of 76 single copy marker genes, the Chloroflexi scaffolds are predicted to represent 24, 24, and 4 Chloroflexi genomes, respectively (average values, Additional file 1: Table S4). Metabolic genes for processes of interest were tabulated for the three depths (Additional file 1: Table S4) and pertinent trends are described below.

Across the Chloroflexi genomic content, both predicted PFOR and PDH complexes for converting pyruvate to acetyl-CoA were identified, with a higher abundance of the typically respiratory-associated PDH complex identified from the 5 m sample. Given the abundance patterns of the Chloroflexi (Figure 2), it is possible this reflects differences in respiratory and fermentative lifestyles across the different clades of Chloroflexi, with members of the Anaerolineae that utilize PDH more abundant in the 5 m depth and the fermentative GIF9 and other lineages more abundant in the shallower sample. Similarly, the 5 m sample contains a high number of predicted cytochrome *c* family proteins, as well as cytochrome *c* oxidases, compared to the 4 m sample.

Respiratory metabolism across the sediment Chloroflexi is limited to the reduction of oxygen and possibly nitrogen species, with no key functional genes for sulfate or sulfide respiration detected. Genes encoding the catalytic subunits of a nitrate reductase (NarG) and a nitrite/nitrate oxidoreductase (NxrA) were identified in each of the 5 m and 6 m samples (Additional file 1: Figure S8) indicating nitrate respiration and nitrification may be encoded, though rarely, in genomes from sediment Chloroflexi. Only the 5 m sample’s *nxrA* gene is accompanied by any associated subunit genes (*nxrBG*). Nitrite reductases were more common, including 10 predicted copper-containing nitrite reductases and eight predicted NrfA (cytochrome c552) nitrite reductases (Additional file 1: Table S4). No genes associated with nitrogen fixation were present.

In keeping with the metabolisms elucidated from the three curated genomes, genes for glycolysis, sugar inter-conversions, and plant compound degradation genes (for example, alpha-amylase, beta-glucosidase, glycoside hydrolase) are present in numbers equivalent to the expected number of organisms. Genes for acetylene hydratase-like and predicted pyrogallol hydroxytransferases were identified on Chloroflexi scaffolds from the 4 and 5 m samples. These data suggest that utilization of plant polymers may be widespread across the Chloroflexi phylum, perhaps explaining their cosmopolitan distribution in sediment systems (Additional file 1: Figure S8). TCA cycle genes were identified, but at lower levels, indicating partial and/or non-functional TCA cycles may be common. A single citrate lyase, indicative of a reverse TCA cycle, was identified on a genome fragment recovered from the 4 m sample.

The key genes for butanoate, lactate, and alcohol fermentation were not found in the sediment Chloroflexi genomes (see Additional file 2). The acetate synthesis pathway via acetate kinase and phosphate acetyltransferase was also not present. However, 12 ADP-dependent acetyl-CoA synthetases were identified, implying the same acetogenesis mechanism described in the curated genomes above is likely present within other Chloroflexi at this site.

### Boundaries on obligate organohalide respiration

RBG-1351 and RBG-2 represent the current nearest genomic neighbors to the Dehalococcoidia (Figure 2), a class notable for obligate organohalide respiration on chlorinated compounds. The Dehalococcoidia utilize reductive dehalogenases for this process: strictly anaerobic, corrinoid co-factor requiring enzymes with varied substrate specificities [93-96]. RBG-1351 and RBG-2 do not contain predicted reductive dehalogenases, and as such, represent new phylogenetic boundaries to the breadth of organohalide respiration within the phylum Chloroflexi.

In direct contrast to the Dehalococcoidia, RBG-2 contains a gene for a predicted haloacid dehalogenase (HAD) of the haloalkane dehalogenase type: aerobic enzymes that catalyze dehalogenation of chlorinated and brominated substrates (Figure 3) [97,98]. The haloalkane and reductive dehalogenase families are not homologous; shared dehalogenase activity evolved independently [99]. The HAD of RBG-2 may confer the ability to utilize chlorinated and brominated compounds, which occur naturally in sediments [100].

Beyond the absence of reductive dehalogenases in RBG-2 and RBG-1351, reductive dehalogenase genes are present in a few of the genomes of other Chloroflexi in the sediment. Interestingly, only five of the 82 predicted reductive dehalogenases in the entire sediment genomic dataset were identified from Chloroflexi scaffolds. Four of the five Chloroflexi reductive dehalogenases are most closely related to a predicted protein from the Deltaproteobacterium *Desulfobacula toluolica* Tol2, an uncharacterized reductive dehalogenase of unknown substrate range (Additional file 1: Figure S9). The last Chloroflexi reductive dehalogenase falls in a deep-branching group of uncharacterized proteins at the base of the Dehalococcoidia-only clade (Additional file 1: Figure S9). This finding indicates a potential role for some, but not many, Chloroflexi in organohalide respiration in the sediment.

## Conclusions

This study indicates that Chloroflexi can be abundant in sediment and involved in carbon cycling in the subsurface. The Chloroflexi sampled here broadly span the Chloroflexi phylogenetic diversity. The genomes are not closely related to one another, nor to organisms with sequenced genomes (Figure 2, Additional file 1: Figure S1) a finding that highlights the biological novelty of sediment. Different lineages have different abundance patterns across depth in the sediment (Figure 2). These patterns may reflect somewhat different niches for the clades. The distribution does not indicate increasing importance of anaerobic metabolism with increasing depth (and distance below the water table), probably an indication of small-scale heterogeneity (for example, in organic carbon content or groundwater flow pathways) in the sediment.

Three draft genomes were curated from the metagenomic data. Two represent anaerobic organisms within Classes sister to the Dehalococcoidia. Both are predicted to be homoacetogens, utilizing a mechanism of acetate formation less common in bacteria. The complete Wood-Ljungdahl pathway for carbon fixation present on these two genomes (RBG-2 and RBG-1351) has not previously been described in the Chloroflexi, though incomplete pathways are present on *Dehalococcoides* and *Thermomicrobium* genomes [14,19]. The third genome, RBG-9, is predicted to belong to a facultative anaerobic, non-motile organism, possibly forming pigmented filaments in the subsurface. RBG-9 is predicted to respire sugars using oxygen as a terminal electron acceptor, and ferment sugars and amino acids in anaerobic conditions.

We analyzed all Chloroflexi-affiliated metagenome sequence information in order to elucidate the potential roles for Chloroflexi in subsurface carbon cycling. The general metabolic profile corresponds to the genomic potential inferred from the three reconstructed genomes, and thus serves to place the detailed, genome-specific analyses in broader context. Overall, the Chloroflexi are predicted to degrade plant compounds, with pathways for the degradation of cellulose, starch, long-chain sugars, and pyrogallol commonly identified. Additionally, the Chloroflexi appear to utilize oxidative phosphorylation and/or acetate fermentation for heterotrophic growth. We identified no evidence for a role for Chloroflexi in sulfur cycling. Some are inferred to function in nitrite and, less commonly, nitrate reduction. Unlike the sediment-associated Dehalococcoidia, organohalide respiration does not appear to be a major metabolic lifestyle for these Chloroflexi. Evidence for both anaerobic and aerobic mechanisms of energy generation suggests the Chloroflexi are able to adapt to the changing redox conditions of the aquifer. Within the broader microbial subsurface community, the Chloroflexi likely compete for labile carbon, degrading starch, sugars, and peptides; and provide organic acids to other organisms in the sediment (for example, acetoclastic methanogens and metal-respiring bacteria). Beyond the Rifle aquifer system, the predicted functions for the Chloroflexi are widely applicable to sediment environments, and may explain the presence of members of this phylum in diverse subsurface locations.

## Availability of supporting data

The sequence datasets supporting the results of this article are available in the ggKbase repository (http://ggkbase.berkeley.edu/rbg/organisms). All predicted gene and protein sequences are in process of deposition in the NCBI sequence database under the existing BioProjectID PRJNA167727.

## Competing interests

The authors declare that they have no competing interests.

## Authors’ contributions

JFB and LAH conceived of the project. KHW managed sample collection. KRF conducted nucleic acid extractions. SGT supervised raw sequence data generation. BCT conducted metagenome assembly and annotation. BCT, JFB, IS, and LAH conducted genome curation. LAH, CJC, KCW, and JFB worked on genome and pathway analysis. LAH and JFB wrote the manuscript. All authors read and approved the final manuscript.

## Supplementary Material

Additional file 1 **Supplemental tables and figures.** Supplemental Tables S1-4, supplemental Figures S1-S9. Click here for file

Additional file 2 **Supplemental text.** Supplemental notes on functions discussed in the main text as well as further information on the three Chloroflexi draft genomes (for example, oxygen tolerance, amino acid biosynthesis, and mobile element signatures). Click here for file
