# Supplementary material for: Community genomic analyses constrain the distribution of metabolic traits across the Chloroflexi phylum and indicate roles in sediment carbon cycling
Source: Microbiome. 2013 Aug 5;1:22. doi: 10.1186/2049-2618-1-22 (PMC3971608; doi:10.1186/2049-2618-1-22)
Supplement: Additional file 1 — Supplemental tables and figures. Supplemental Tables S1-4, supplemental Figures S1-S9. [file 2049-2618-1-22-S1.pdf]

Community genomic analyses constrain the distribution of metabolic traits across the Chloroflexi phylum and indicate roles in sediment carbon cycling

Laura A. Hug, Cindy J. Castelle, Kelly C. Wrighton, Brian C. Thomas, Itai Sharon, Kyle R. Frischkorn, Kenneth H. Williams, Susannah G. Tringe, and Jillian F. Banfield

Supplemental Figures and Tables

Table S1: Marker gene presence/absence used to determine RBG-2 genome completion. Annotations are from scaffolds greater than 2,500 bp in length. The genus and phylum of the closest BLAST match for each gene are listed. Three of the 76 marker genes are present twice: tRNA synthetases Cys, His, and Thr. The remainder of the marker genes are present in the genome in single copy.

GENE NAME	#	Gene ID	Taxonomic Affiliation	PHYLUM
preprotein translocase secG	1	scaffold_1_99	*Dehalococcoides*	Chloroflexi
ribonuclease P protein	1	scaffold_1_211	*Dehalococcoides*	Chloroflexi
recombinase A	1	scaffold_3_25	*Dehalococcoides*	Chloroflexi
DNA gyrase A	1	scaffold_7_21	*Dehalococcoides*	Chloroflexi
DNA gyrase B	1	scaffold_4_145	*Dehalococcoides*	Chloroflexi
DNA RNA polymerase beta	1	scaffold_2_61	*Dehalococcoides*	Chloroflexi
elongation factor P	1	scaffold_1_179	*Syntrophothermus*	Firmicutes
5S rRNA	0			
16S rRNA	1	scaffold_14_L1	GIF9 uncultured bacteria	Chloroflexi
23S rRNA	1	scaffold_11_L1	*Dehalococcoides*	Chloroflexi
ribosomal protein L1	1	scaffold_8_91	*Dehalococcoides*	Chloroflexi
ribosomal protein L2	1	scaffold_5_169	*Thermomicrobium*	Chloroflexi
ribosomal protein L3	1	scaffold_5_172	*Thermobaculum*	Bacteria
ribosomal protein L4	1	scaffold_5_171	*Dehalococcoides*	Chloroflexi
ribosomal protein L5	1	scaffold_5_160	*Thermoanaerobacterium*	Firmicutes
ribosomal protein L6	1	scaffold_5_158	*Caldalkalibacillus*	Firmicutes
ribosomal protein L7/L12	1	scaffold_6_53	*Thermomicrobium*	Chloroflexi
ribosomal protein L9	1	scaffold_3_123	*Thermobaculum*	Bacteria
ribosomal protein L10	1	scaffold_6_54	*Dehalogenimonas*	Chloroflexi
ribosomal protein L11	1	scaffold_8_92	*Carboxydothermus*	Firmicutes
ribosomal protein L13	1	scaffold_5_144	*Thermobispora*	Actinobacteria
ribosomal protein L14	1	scaffold_5_162	*Halothermothrix*	Firmicutes
ribosomal protein L15	1	scaffold_5_155	*Dehalogenimonas*	Chloroflexi
ribosomal protein L16	1	scaffold_5_165	*Dehalogenimonas*	Chloroflexi
ribosomal protein L17	1	scaffold_5_146	*Geobacter*	Proteobacteria
ribosomal protein L18	1	scaffold_5_157	*Coprothermobacter*	Firmicutes
ribosomal protein L19	1	scaffold_7_39	*Dehalogenimonas*	Chloroflexi
ribosomal protein L20	1	scaffold_8_114	*Dehalococcoides*	Chloroflexi
ribosomal protein L21	1	scaffold_4_159	*Dehalococcoides*	Chloroflexi
ribosomal protein L22	1	scaffold_5_167	*Dehalococcoides*	Chloroflexi
ribosomal protein L23	1	scaffold_5_170	*Dehalogenimonas*	Chloroflexi
ribosomal protein L24	1	scaffold_5_161	*Dehalococcoides*	Chloroflexi
ribosomal protein L25	1	scaffold_3_99	*Dehalogenimonas*	Chloroflexi

ribosomal protein L27	1	scaffold_4_160	*Leptospira*	Spirochaetes
ribosomal protein L28	1	scaffold_4_25	*Dehalococcoides*	Chloroflexi
ribosomal protein L29	1	scaffold_5_164	*Streptomyces*	Actinobacteria
ribosomal protein S1	0	n/a	n/a	
ribosomal protein S2	1	scaffold_8_103	*Dehalogenimonas*	Chloroflexi
ribosomal protein S3	1	scaffold_5_166	*Dehalogenimonas*	Chloroflexi
ribosomal protein S4	1	scaffold_5_148	*Dehalococcoides*	Chloroflexi
ribosomal protein S5	1	scaffold_5_156	*Halanaerobium*	Firmicutes
ribosomal protein S6	1	scaffold_1_213	*Moorella*	Firmicutes
ribosomal protein S7	1	scaffold_5_175	Candidatus *Odyssella*	Proteobacteria
ribosomal protein S8	1	scaffold_5_159	*Roseiflexus*	Chloroflexi
ribosomal protein S9	1	scaffold_5_143	*Thermaerobacter*	Firmicutes
ribosomal protein S10	1	scaffold_5_173	*Parvibaculum*	Proteobacteria
ribosomal protein S11	1	scaffold_5_149	*Dehalococcoides*	Chloroflexi
ribosomal protein S12	1	scaffold_5_176	*Dehalogenimonas*	Chloroflexi
ribosomal protein S13	1	scaffold_5_150	*Ktedonobacter*	Chloroflexi
ribosomal protein S14	1	scaffold_5_LAH1	*Dehalogenimonas*	Chloroflexi
ribosomal protein S15	1	scaffold_2_38	*Mahella*	Firmicutes
ribosomal protein S16	1	scaffold_5_85	*Thermosediminibacter*	Firmicutes
ribosomal protein S17	1	scaffold_5_163	*Ilyobacter*	Fusobacteria
ribosomal protein S18	1	scaffold_1_LAH1	*Dehalogenimonas*	Chloroflexi
ribosomal protein S19	1	scaffold_5_168	*Syntrophothermus*	Firmicutes
ribosomal protein S20	1	scaffold_4_142	*Anaerolinea*	Chloroflexi
tRNA synthetase ala	1	scaffold_1_197	*Dehalococcoidia*	Chloroflexi
tRNA synthetase arg	1	scaffold_4_28	*Dehalococcoidia*	Chloroflexi
tRNA synthetase asp	1	scaffold_1_185	*Oscillochloris*	Chloroflexi
tRNA synthetase asn	0			
tRNA synthetase cys	2	scaffold_7_80, scaffold_11_35	*Dehalococcoides, Archaeoglobus*	Chloroflexi, Archaea
tRNA synthetase gln	0			
tRNA synthetase glu	1	scaffold_1_137	*Dehalococcoidia*	Chloroflexi
tRNA synthetase gly	1	scaffold_1_282	*Dehalococcoidia*	Chloroflexi
tRNA synthetase his	2	scaffold_4_144, scaffold_7_66	*Dehalococcoidia*	Chloroflexi
tRNA synthetase ile	1	scaffold_1_206	*Dehalococcoidia*	Chloroflexi
tRNA synthetase leu	1	scaffold_1_135	*Dehalococcoidia*	Chloroflexi
tRNA synthetase lys	1	scaffold_2_150	*Dehalococcoidia*	Chloroflexi
tRNA synthetase met	2	scaffold_1_221, scaffold_21_1	*DehalococcoidiaDehalococcoidia*	Chloroflexi, Chloroflexi
tRNA synthetase phe	1	scaffold_8_112/113 (split gene)	*Dehalococcoidia*	Chloroflexi
tRNA synthetase pro	1	scaffold_8_111	*Dehalococcoidia*	Chloroflexi
tRNA synthetase ser	1	scaffold_2_148	*Thermomicrobium*	Chloroflexi
tRNA synthetase thr	2	scaffold_8_117,	*Dehalococcoides,*	Chloroflexi,

		scaffold_9_78	*Methanohalophilus*	Archaea
tRNA synthetase tryp	1	scaffold_5_140	*Dehalococcoidia*	Chloroflexi
tRNA synthetase tyr	1	scaffold_1_240	*Dehalococcoidia*	Chloroflexi
tRNA synthetase val	1	scaffold_7_4	*Dehalococcoidia*	Chloroflexi

Table S2: Marker gene presence/absence used to determine RBG-9 genome completion. Annotations are from scaffolds greater than 2,500 bp in length. The genus and phylum of the closest BLAST match for each gene are listed. DNA gyrase A and tRNA synthetase Pro are duplicated, while Recombinase A is present as a fragmented gene at the ends of scaffolds. The remainder of the marker genes is present in the genome in single copy.

GENE NAME	#	Gene ID	Taxonomic Affiliation	Phylum
preprotein translocase secG	1	RBG9_12_32	*Anaerolinea*	Chloroflexi
ribonuclease P protein	1	RBG9_17_40	*Anaerolinea*	Chloroflexi
recombinase A	3	RBG9_63_133/ RBG9_4_1/ RBG9_33_17	*Rhizobium*/ *Anaerolinea*/ *Anaerolinea*	Alphaproteobacteria/ Chloroflexi/ Chloroflexi
DNA gyrase A	2	RBG9_41_166/ RBG9_4_60	*Neisseria* / *Anaerolinea*	Betaproteobacteria/ Chloroflexi
DNA gyrase B	1	RBG9_17_46	*Anaerolinea*	Chloroflexi
DNA RNA polymerase beta	1	RBG9_63_124	*Anaerolinea*	Chloroflexi
elongation factor P	1	RBG9_59_186	uncultured bacterium	Chloroflexi
5S rRNA	0			
16S rRNA	1	RBG9_scaffold_43_4861_6023	Anaerolinea	Chloroflexi
23S rRNA	1	RBG9_23S_rRNA	*Anaerolinea*	Chloroflexi
ribosomal protein L1	1	RBG9_46_159	*Anaerolinea*	Chloroflexi
ribosomal protein L2	1	RBG9_63_115	*Anaerolinea*	Chloroflexi
ribosomal protein L3	1	RBG9_63_118	*Anaerolinea*	Chloroflexi
ribosomal protein L4	1	RBG9_63_117	uncultured bacterium	Chloroflexi
ribosomal protein L5	1	RBG9_63_106	uncultured bacterium	Chloroflexi
ribosomal protein L6	1	RBG9_63_103	*Anaerolinea*	Chloroflexi
ribosomal protein L7/L12	1	RBG9_46_157	*Anaerolinea*	Chloroflexi
ribosomal protein L9	1	RBG9_45_23	*Anaerolinea*	Chloroflexi
ribosomal protein L10	1	RBG9_46_158	*Phascolarctobacterium*	Firmicutes
ribosomal protein L11	1	RBG9_46_160	*Anaerolinea*	Chloroflexi
ribosomal protein L13	1	RBG9_63_89	*Desulfohalobium*	deltaProteobacteria

ribosomal protein L14	1	RBG9_63_108	*Anaerolinea*	Chloroflexi
ribosomal protein L15	1	RBG9_63_100	*Anaerolinea*	Chloroflexi
ribosomal protein L16	1	RBG9_63_111	*Thermoanaerobacter*	Firmicutes
ribosomal protein L17	1	RBG9_63_91	*Anaerolinea*	Chloroflexi
ribosomal protein L18	1	RBG9_63_102B	*Anaerolinea*	Chloroflexi
ribosomal protein L19	1	RBG9_41_168	uncultured bacterium	Chloroflexi
ribosomal protein L20	1	RBG9_25_156	*Paenibacillus*	Firmicutes
ribosomal protein L21	1	RBG9_57_66	*Moorella*	Firmicutes
ribosomal protein L22	1	RBG9_63_113	*Anaerolinea*	Chloroflexi
ribosomal protein L23	1	RBG9_63_116	uncultured bacterium	Chloroflexi
ribosomal protein L24	1	RBG9_63_107	*Anaerolinea*	Chloroflexi
ribosomal protein L25	1	RBG9_57_46	uncultured Chloroflexi bacterium	Chloroflexi
ribosomal protein L27	1	RBG9_57_67	*Acaryochloris*	Cyanobacteria
ribosomal protein L28	1	RBG9_59_170	uncultured bacterium	Chloroflexi
ribosomal protein L29	1	RBG9_63_110	*Streptomyces*	Actinobacteria
ribosomal protein S1	1	RBG9_14_55	*Caldilinea*	Chloroflexi
ribosomal protein S2	1	RBG9_41_77	*Anaerolinea*	Chloroflexi
ribosomal protein S3	1	RBG9_63_112	*Anaerolinea*	Chloroflexi
ribosomal protein S4	1	RBG9_63_93	*Anaerolinea*	Chloroflexi
ribosomal protein S5	1	RBG9_63_102	*Anaerolinea*	Chloroflexi
ribosomal protein S6	1	RBG9_12_42	*Anaerolinea*	Chloroflexi
ribosomal protein S7	1	RBG9_63_122	*Anaerolinea*	Chloroflexi
ribosomal protein S8	1	RBG9_63_104	*Anaerolinea*	Chloroflexi
ribosomal protein S9	1	RBG9_63_88	*Anaerolinea*	Chloroflexi
ribosomal protein S10	1	RBG9_63_119	uncultured bacterium	Chloroflexi
ribosomal protein S11	1	RBG9_63_94	*Anaerolinea*	Chloroflexi
ribosomal protein S12	1	RBG9_63_123	*Anaerolinea*	Chloroflexi
ribosomal protein S13	1	RBG9_63_95	*Anaerolinea*	Chloroflexi
ribosomal protein S14	1	RBG9_63_105	*Anaerolinea*	Chloroflexi
ribosomal protein S15	1	RBG9_14_46	*Anaerolinea*	Chloroflexi
ribosomal protein S16	1	RBG9_59_155	*Anaerolinea*	Chloroflexi
ribosomal protein S17	1	RBG9_63_109	*Anaerolinea*	Chloroflexi
ribosomal protein S18	1	RBG9_12_40	uncultured bacterium	Chloroflexi
ribosomal protein S19	1	RBG9_63_114	uncultured bacterium	Chloroflexi
ribosomal protein S20	1	fragmented	uncultured Chloroflexi	Chloroflexi
tRNA synthetase ala	1	RBG9_59_144	*Anaerolinea*	Chloroflexi
tRNA synthetase arg	1	RBG9_57_86	uncultured Chloroflexi	Chloroflexi
tRNA synthetase asp	1	RBG9_17_97	*Chloroflexus*	Chloroflexi
tRNA synthetase asn	0			
tRNA synthetase cys	1	RBG9_65_87	*Anaerolinea*	Chloroflexi
tRNA synthetase gln	0			
tRNA synthetase glu	1	RBG9_12_25	*Anaerolinea*	Chloroflexi
tRNA synthetase gly	1	RBG9_41_48	*Anaerolinea*	Chloroflexi

tRNA synthetase his	1	RBG9_56_61	*Anaerolinea*	Chloroflexi
tRNA synthetase ile	1	RBG9_56_8	*Thermobaculum*	unclassified Bacteria
tRNA synthetase leu	1	RBG9_66_159	*Anaerolinea*	Chloroflexi
tRNA synthetase lys	1	RBG9_64_158	*Anaerolinea*	Chloroflexi
tRNA synthetase met	1	RBG9_7_105	*Sphaerobacter*	Chloroflexi
tRNA synthetase phe	1	RBG9_7_2 (alpha)/RBG9_7_73(beta)	*Ornithinibacillus* / *Anaerolinea*	Firmicutes/ Chloroflexi
tRNA synthetase pro	2	RBG9_7_71/RBG9_12_25	*Caldilinea* / *Anaerolinea*	Chloroflexi/ Chloroflexi
tRNA synthetase ser	1	RBG9_25_56	*Roseiflexus*	Chloroflexi
tRNA synthetase thr	1	RBG9_46_116	*Spirochaeta*	Spirochaeta
tRNA synthetase tryp	1	RBG9_8_26	*Spirochaeta*	Spirochaeta
tRNA synthetase tyr	1	RBG9_59_146	*Caldilinea*	Chloroflexi
tRNA synthetase val	1	RBG9_59_50	*Anaerolinea*	Chloroflexi

Table S3: Marker gene presence/absence used to determine RBG-1351 genome completion. Annotations are from scaffolds greater than 2,500 bp in length. The genus and phylum of the closest BLAST match for each gene are listed. tRNA synthetases Gly and His are duplicated. The remainder of the marker genes is present in the genome in single copy.

GENE NAME	#	Gene ID	Taxonomic Affiliation	Phylum
preprotein translocase secG	1	RBG1351_10_49	*Dehalococcoides*	Chloroflexi
ribonuclease P protein	1	RBG1351_13_31	*Caldilinea*	Chloroflexi
recombinase A	1	RBG1351_24_6	*Dehalococcoides*	Chloroflexi
DNA gyrase A	1	RBG1351_23_13	*Dehalococcoides*	Chloroflexi
DNA gyrase B	1	RBG1351_16_2	*Dehalococcoides*	Chloroflexi
DNA RNA polymerase beta	1	RBG1351_8_44	*Dehalococcoides*	Chloroflexi
elongation factor P	1	RBG1351_4_6	*Dehalococcoides*	Chloroflexi
5S rRNA	0			
16S rRNA	0			
23S rRNA	0			
ribosomal protein L1	1	RBG1351_8_17	*Dehalococcoides*	Chloroflexi
ribosomal protein L2	1	RBG1351_17_21	*Dehalococcoides*	Chloroflexi
ribosomal protein L3	1	RBG1351_17_18	*Dehalogenimonas*	Chloroflexi
ribosomal protein L4	1	RBG1351_17_19	*Dehalococcoides*	Chloroflexi
ribosomal protein L5	1	RBG1351_17_30	*Desulfotomaculum*	Firmicutes
ribosomal protein L6	1	RBG1351_17_32	*Dehalococcoides*	Chloroflexi
ribosomal protein L7/L12	1	RBG1351_8_19	*Thermoanaerobacterium*	Firmicutes
ribosomal protein L9	1	RBG1351_27_18	*Thermobaculum*	unclassified

				Bacteria
ribosomal protein L10	1	RBG1351_8_18	*Dehalococcoides*	Chloroflexi
ribosomal protein L11	1	RBG1351_8_16	*Carboxydothermus*	Firmicutes
ribosomal protein L13	1	RBG1351_17_48	*Thermobaculum*	unclassified Bacteria
ribosomal protein L14	1	RBG1351_17_28	*Coprococcus*	Firmicutes
ribosomal protein L15	1	RBG1351_17_36	*Dehalococcoides*	Chloroflexi
ribosomal protein L16	1	RBG1351_17_25	*Dehalogenimonas*	Chloroflexi
ribosomal protein L17	1	RBG1351_17_46	*Geobacter*	Deltaproteo bacteria
ribosomal protein L18	1	RBG1351_17_33	*Dehalogenimonas*	Chloroflexi
ribosomal protein L19	1	RBG1351_9_10	*Dehalogenimonas*	Chloroflexi
ribosomal protein L20	1	RBG1351_12_6	*Dehalococcoides*	Chloroflexi
ribosomal protein L21	1	RBG1351_2_47	*Dehalococcoides*	Chloroflexi
ribosomal protein L22	1	RBG1351_17_23	*Dehalogenimonas*	Chloroflexi
ribosomal protein L23	1	RBG1351_17_20	*Dehalococcoides*	Chloroflexi
ribosomal protein L24	1	RBG1351_17_29	*Dehalococcoides*	Chloroflexi
ribosomal protein L25	0			
ribosomal protein L27	1	RBG1351_2_48	*Dehalococcoides*	Chloroflexi
ribosomal protein L28	1	RBG1351_14_16	*Dehalogenimonas*	Chloroflexi
ribosomal protein L29	1	RBG1351_17_26	*Dehalococcoides*	Chloroflexi
ribosomal protein S1	0			
ribosomal protein S2	1	RBG1351_11_7	*Dehalococcoides*	Chloroflexi
ribosomal protein S3	1	RBG1351_17_27	*Herpetosiphon*	Chloroflexi
ribosomal protein S4	1	RBG1351_17_44	*Dehalogenimonas*	Chloroflexi
ribosomal protein S5	1	RBG1351_17_34	*Dehalococcoides*	Chloroflexi
ribosomal protein S6	1	RBG1351_13_28	*Dehalogenimonas*	Chloroflexi
ribosomal protein S7	1	RBG1351_17_15	*Sphaerobacter*	Chloroflexi
ribosomal protein S8	1	RBG1351_17_31	*Dehalococcoides*	Chloroflexi
ribosomal protein S9	1	RBG1351_17_49	*Dehalococcoides*	Chloroflexi
ribosomal protein S10	1	RBG1351_17_17	*Dehalococcoides*	Chloroflexi
ribosomal protein S11	1	RBG1351_17_43	*Dehalococcoides*	Chloroflexi
ribosomal protein S12	1	RBG1351_17_14	*Dehalococcoides*	Chloroflexi
ribosomal protein S13	1	RBG1351_17_42	*Dehalococcoides*	Chloroflexi
ribosomal protein S14	1	RBG1351_17_30 B	*Acetivibrio*	Firmicutes
ribosomal protein S15	1	RBG1351_32_10	*Caloramaor*	Firmicutes
ribosomal protein S16	0			
ribosomal protein S17	1	RBG1351_17_27	*Herpetosiphon*	Chloroflexi
ribosomal protein S18	1	RBG1351_13_30	*Dehalococcoides*	Chloroflexi
ribosomal protein S19	1	RBG1351_17_22	*Dehalogenimonas*	Chloroflexi
ribosomal protein S20	1	RBG1351_10_15	*Nitrolancetus*	Chloroflexi
tRNA synthetase ala	1	RBG1351_8_51	*Dehalococcoides*	Chloroflexi
tRNA synthetase arg	0			
tRNA synthetase asp	1	RBG1351_2_28	*Dehalococcoides*	Chloroflexi
tRNA synthetase asn	0			

tRNA synthetase cys	1	RBG1351_3_24	*Dehalogenimonas*	Chloroflexi
tRNA synthetase gln	0			
tRNA synthetase glu	1	RBG1351_1_3	*Dehalococcoides*	Chloroflexi
tRNA synthetase gly	2	RBG1351_5_28/<br>RBG1351_10_8	*Dehalococcoides*/<br>*Methylomicrobium*	Chloroflexi/<br>Gammaprot eobacteria
tRNA synthetase his	3	RBG1351_10_19/<br>RBG1351_21_29/<br>RBG1351_46_1	*Dehalococcoides/*<br>*Dehalococcoides/*<br>*Dehalococcoides*	
tRNA synthetase ile	1	RBG1351_13_36	*Dehalococcoides*	Chloroflexi
tRNA synthetase leu	1	RBG1351_9_22	*Dehalogenimonas*	Chloroflexi
tRNA synthetase lys	1	RBG1351_18_21	*Dehalococcoides*	Chloroflexi
tRNA synthetase met	1	RBG1351_12_38	*Dehalococcoides*	Chloroflexi
tRNA synthetase phe	2	RBG1351_12_7/<br>RBG1351_12_8	*Dehalogenimonas* /<br>*Dehalogenimonas*	Chloroflexi/<br>Chloroflexi
tRNA synthetase pro	1	RBG1351_12_15	*Dehalococcoides*	Chloroflexi
tRNA synthetase ser	1	RBG1351_18_19	*Dehalococcoides*	Chloroflexi
tRNA synthetase thr	1	RBG1351_12_3	*Dehalogenimonas*	Chloroflexi
tRNA synthetase tryp	1	RBG1351_13_41	*Halothermothrix*	Firmicutes
tRNA synthetase tyr	1	RBG1351_11_43	*Dehalococcoides*	Chloroflexi
tRNA synthetase val	1	RBG1351_28_20	*Dehalogenimonas*	Chloroflexi

Table S4: Counts of metabolic genes of interest and single copy genome-completeness marker genes identified from the Chloroflexi scaffolds. Gene counts do not include the three curated draft genomes.

METABOLIC	GENE NAME	4m	5m	6m
**Cellulose degradation**	beta-glucosidase	20	45	13
	cellulase	7	6	4
	endoglucanase/ aminopeptidase	23	40	13
	cellobiosidase	0	1	0
	glycoside hydrolase	32	38	4
	beta-mannosidase	2	9	2
	glucoamylase	0	0	0
	glycosyl hydrolase	18	22	2
**Amylotic degradation**	alpha amylase	23	38	11
	glucanase	1	0	0
	isoamylase	0	0	0
	malto-oligosyltrehalose trehalohydrolase	0	3	0
	amylopullulanase	1	0	0
**Chitin**	chitin deacetylase	0	3	0
	glucan endo-1,3-beta-D-glucosidase	0	4	0
**Sugar Interconversions**	sugar kinase	7	2	0
	alpha-glucan phosphorylase	1	4	0
	adenosine kinase	6	17	6
	cytidyltransferase	1	7	2
	acetylglucosamine 2-epimerase	18	25	4
	triosephosphate isomerase	26	22	6
	mannose-1-phosphate guanylyltransferase	18	26	1
	phosphomannomutase	16	11	1
	beta phosphoglucomutase	3	4	1
	glycogen debranching enzyme	1	4	1
	pectin lyase	0	0	0
**Glycolysis**	hexokinase	0	0	0
	pyruvate kinase	18	18	2
	phosphoglucose isomerase	27	23	5
	phosphofructokinase	18	33	11
	fructose bisphosphate aldolase	9	12	7
	triose phosphate isomerase	26	22	6
	glyceraldehyde phosphate	27	31	2

	dehydrogenase			
	phosphoglycerate kinase	34	22	5
	phosphoglycerate mutase	77	65	18
	enolase	25	26	2
**TCA cycle**	citrate synthase	9	24	5
	aconitate hydratase	6	23	6
	isocitrate dehydrogenase	15	23	9
	2-oxoglutarate dehydrogenase E1	1	2	0
	2-oxoglutarate dehydrogenase E2	9	18	6
	succinyl coA synthetase alpha	11	10	4
	succinyl coA synthetase beta	11	10	3
	succinate dehydrogenase (A-D)	7	7	4
	fumarate hydratase	20	31	11
	malate dehydrogenase	11	17	6
**reverse TCA**	citrate lyase	1	0	1
	fumarate reductase	7	18	3
	oxoglutarate ferredoxin oxidoreductase	15	33	7
**Carbon**	lactate dehydrogenase	1	7	1
	pyruvate carboxylase	3	3	3
	pyruvate decarboxylase	0	0	0
	pyruvate formate lyase	6	0	3
	PFOR alpha	15	7	4
	pyruvate dehydrogenase E1 alpha	17	38	10
	RuBisCo (form IV only)	2	2	0
	pyrogallol hydroxytransferase	1	8	0
	acetylene hydratase (putative)	8	7	0
**CODH/Wood Ljungdahl**	ACS/CODH	5	6	3
	CODH aerobic	1	1	0
	CODH anaerobic not associated with ACS	1	33	6
**Acetogenesis**	ADP-dependent ACS-I	8	4	0
	acetate kinase	0	0	0
	phosphate acetyltransferase	0	0	0
	butyrate kinase	0	0	0
	glucosamine-1-phosphate N-acetyltransferase	27	29	9
	malic enzyme	12	9	1
	long-chain acyl-CoA synthetase	41	39	4

**butanoate**	acetolactate synthase	16	18	2
	short chain enoyl-CoA hydratase	6	29	10
	enoyl-CoA hydratase	40	30	5
	crotonase	0	0	0
	hydroxybutyrl-coA dehydrogenase	22	32	3
**Hydrogen/Proton Motive Force**	hydrogenase (NiFe)	12	19	2
	hydrogenase (FeFe)	19	6	1
	cytochrome *bd* oxidase	7	1	1
	cytochrome *b6*	7	11	0
	cytochrome *b5*	1	3	1
	cytochrome *b*	19	34	7
	cytochrome *b/c*	11	22	5
	cytochrome *c* oxidase (subunit I)	8	16	3
	cytochrome *c*	74	158	42
	cytochrome P450	0	5	2
	ferredoxins	135	140	25
	NADH dehydrogenase A-N	66	118 subunits	13
	formate dehydrogenase A	17	29	5
	pyrophosphatase (*hppA*)	24	27	7
	electron transport complex, *rnfABCDGE*	1	1 (B subunit)	0
**ATP synthase**	F1F0-type A	10	27	7
	F1F0-type alpha	11	31	6
	A1A0-type A subunit	1	2	0
	V1V0-type A subunit	0	0	0
**Nitrate reduction**	nitrate reductase cytochrome c-type subunit (*napB*)	0	0	0
	nitrate reductase (*narG*)	0	1	1
	nitrate reductase (*nxrA*)	0	1	1
	nitrite reductase (cytochrome *cd*)	1	1	0
	nitrite reductase (Cu-containing)	3	7	0
	nitrite reductase (*nirB*)	1	2	0
	cytochrome c nitrite reductase *nrfA/nfrH*	0	8	0
	2-nitropropane dioxygenase	4	4	1
	nitrous oxide reductase	0	1	0
**Nitrogen fixation**	nitrogenase	0	0	0
	dinitrogenase	0	0	0

	nitrogenase iron protein *nifH*	0	0	0
	nifK (beta molyb)	0	0	0
	nifD (alpha molyb)	0	0	0
**Sulfate**	adenylylsulfate reductase (alpha) (*aprA*)	1	1	1
	adenylylsulfate reductase (beta) (*aprB*)	0	0	0
	sulfate adenylyltransferase	3	13	0
	dissimilatory sulfite reductase (*dsrA*)	0	0	0
	dissimilatory sulfite reductase (*dsrB*)	0	0	0
	dissimilatory sulfite reductase (*dsrC* - gamma subunit)	4	3	1
	heterodisulfide reductase A	20	15	1
	heterodisulfide reductase B	4	11	1
	heterodisulfide reductase C	7	9	0
	sulfite oxidase (*yedY* only)	4	10	2
**Other**	ferric reductase	4	6	3
	flagella	14	12 (one operon)	0
	LPS genes (no full pathways)	3	7	0
	peptidoglycan (no full pathways)	2	0	0
	haloacid dehalogenases	14	18	4
	reductive dehalogenases	3	2	0
	selenate reductase	0	0	0
	benzoate/BTEX degradation (no full pathways)	27	39	6
**SINGLE COPY GENES**		**4m**	**5m**	**6m**
	**Average (not including rRNAs)**	23.8	24.4	4.5
	**Standard deviation**	6.8	5.6	2
	**Maximum #**	44	41	10
	**Minimum #**	4	9	2
	preprotein translocase secG	20	19	5
	ribonuclease P protein	22	23	6
	recombinase A	27	28	5
	DNA gyrase A	32	29	5
	DNA gyrase B	18	29	5
	DNA RNA polymerase beta	28	30	10
	elongation factor P	29	23	5
	16S rRNA	12	9	2

	23S rRNA	6	10	3
	5S rRNA	0	5	0
	ribosomal protein L1	17	24	4
	ribosomal protein L2	28	27	4
	ribosomal protein L3	27	26	3
	ribosomal protein L4	28	26	4
	ribosomal protein L5	32	28	3
	ribosomal protein L6	28	29	4
	ribosomal protein L7/L12	14	24	3
	ribosomal protein L9	19	26	3
	ribosomal protein L10	17	24	3
	ribosomal protein L11	17	41	9
	ribosomal protein L13	28	25	4
	ribosomal protein L14	31	27	3
	ribosomal protein L15	26	29	6
	ribosomal protein L16	29	23	2
	ribosomal protein L17	28	24	5
	ribosomal protein L18	28	27	4
	ribosomal protein L19	24	25	2
	ribosomal protein L20	19	19	4
	ribosomal protein L21	19	15	2
	ribosomal protein L22	23	26	3
	ribosomal protein L23	14	25	4
	ribosomal protein L24	30	27	4
	ribosomal protein L25	32	19	4
	ribosomal protein L27	18	16	2
	ribosomal protein L28	3	13	1
	ribosomal protein L29	24	24	2
	ribosomal protein S1	21	37	10
	ribosomal protein S2	26	31	4
	ribosomal protein S3	27	27	3
	ribosomal protein S4	30	27	6
	ribosomal protein S5	30	31	4
	ribosomal protein S6	24	26	9
	ribosomal protein S7	21	26	3
	ribosomal protein S8	30	26	4
	ribosomal protein S9	27	25	4
	ribosomal protein S10	22	23	2
	ribosomal protein S11	27	27	5
	ribosomal protein S12	25	40	6
	ribosomal protein S13	27	27	6
	ribosomal protein S14	11	14	2
	ribosomal protein S15	26	22	3

	ribosomal protein S16	17	23	5
	ribosomal protein S17	25	26	3
	ribosomal protein S18	12	18	8
	ribosomal protein S19	24	25	3
	ribosomal protein S20	13	9	5
	tRNA synthetase ala	21	26	5
	tRNA synthetase arg	20	13	4
	tRNA synthetase asp	22	20	4
	tRNA synthetase asn	4	7	1
	tRNA synthetase cys	33	29	10
	tRNA synthetase gln	8	8	3
	tRNA synthetase glu	4	22	8
	tRNA synthetase gly	19	21	3
	tRNA synthetase his	33	21	5
	tRNA synthetase ile	29	25	5
	tRNA synthetase leu	25	17	5
	tRNA synthetase lys	34	26	4
	tRNA synthetase met	27	29	7
	tRNA synthetase phe	26	19	5
	tRNA synthetase pro	44	32	7
	tRNA synthetase ser	22	22	2
	tRNA synthetase thr	24	19	4
	tRNA synthetase tryp	19	19	3
	tRNA synthetase tyr	21	24	6
	tRNA synthetase val	23	22	4

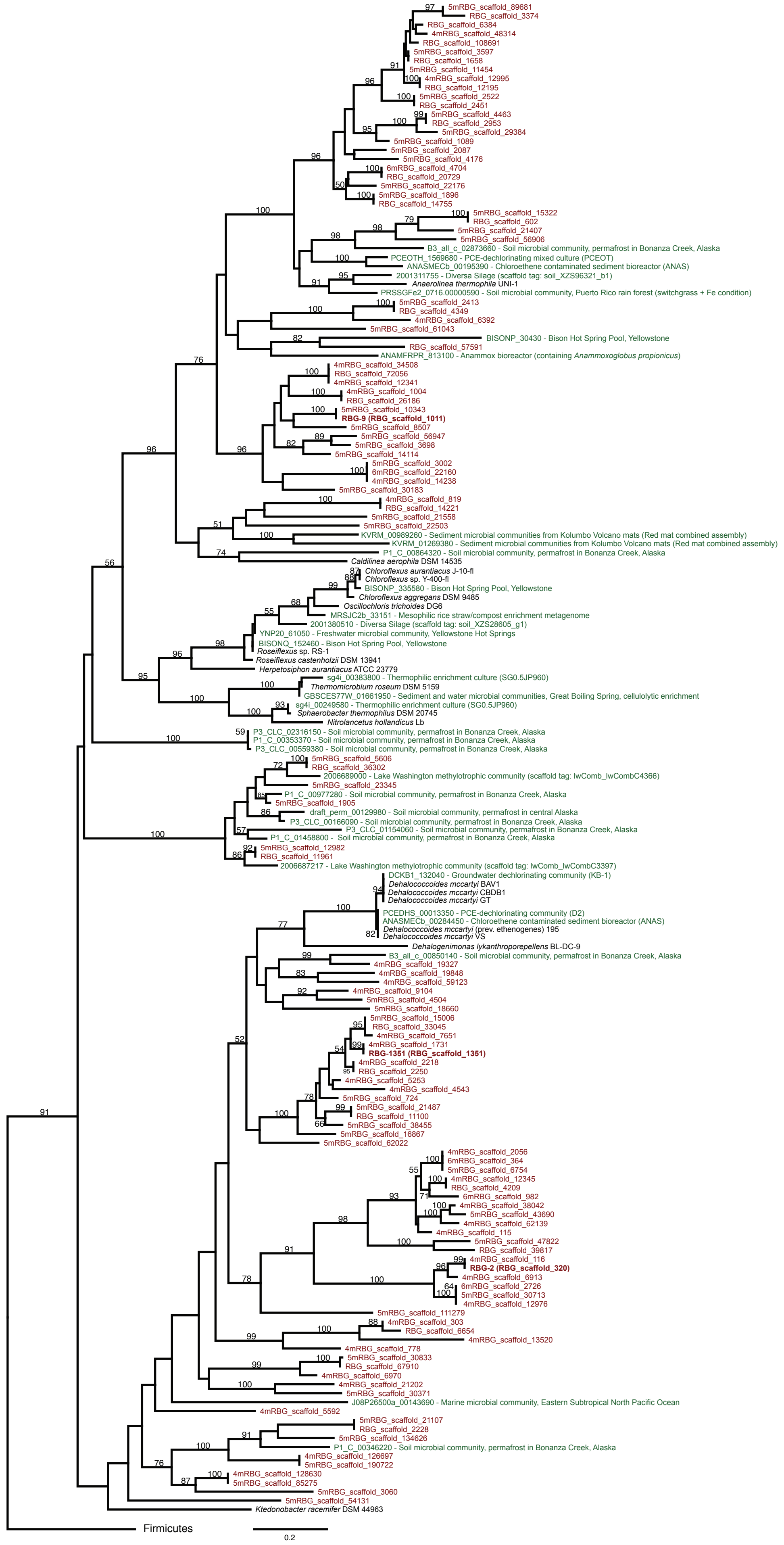

Figure S1: Complete maximum likelihood phylogeny of the Chloroflexi phylum based on an Rps3 protein alignment, as simplified in Figure 2. Bootstrap values greater than 50 are displayed. Rifle-derived RpS3 sequence names are in red, complete genomes in black and environmental sequences mined from NCBI and IMG/M in green. Lineages highlighted in bold represent the three curated genomes. See main text methods for alignment and phylogeny details.

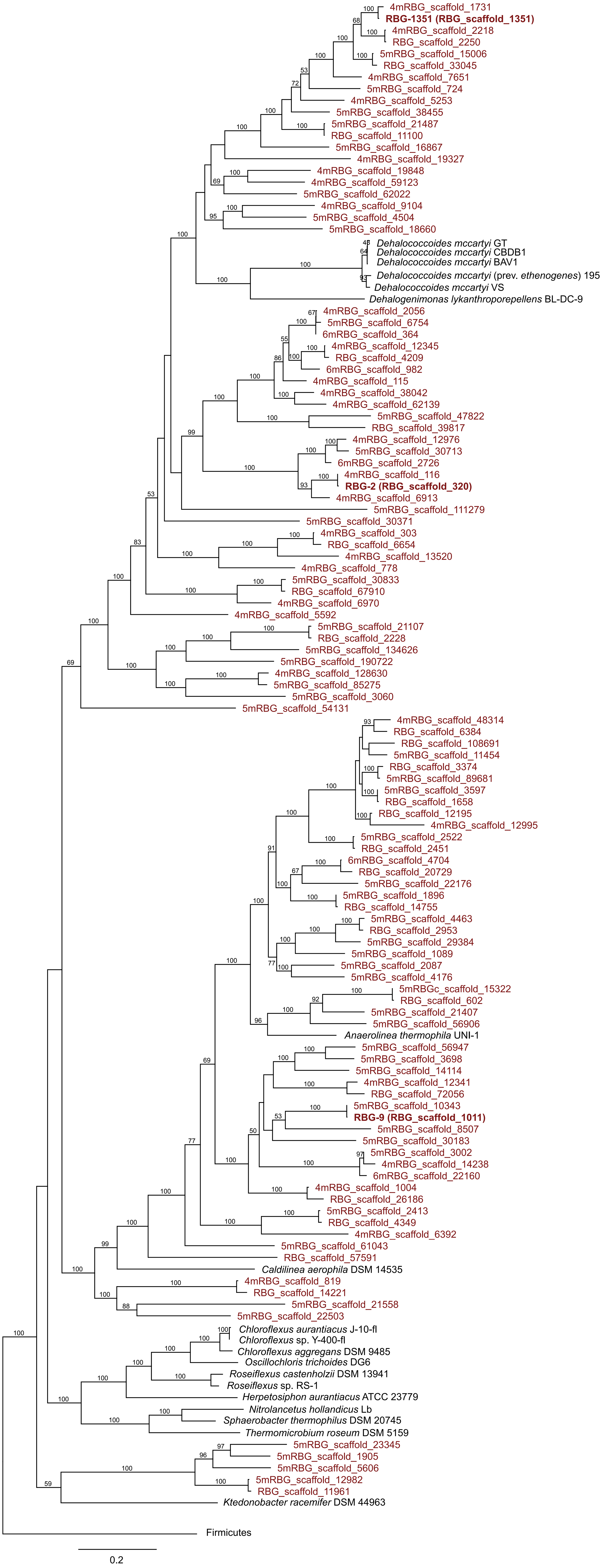

Figure S2: Maximum likelihood phylogeny of the Chloroflexi phylum based on a concatenated alignment of sixteen ribosomal proteins. Rifle Chloroflexi lineages are in red. Lineages highlighted in bold represent the three curated genomes. Bootstrap support values greater than 50 are displayed. See main text methods for alignment and phylogeny details.

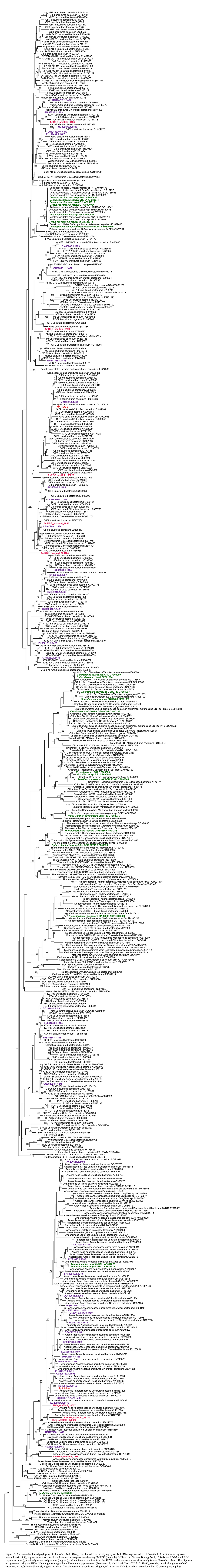

Figure S3: Maximum likelihood phylogeny of Chloroflexi 16S rRNA genes. Included on the phylogeny are 16S rRNA sequences derived from the Rifle sediment metagenome assemblies (in pink), sequences reconstructed from the round one sequence reads using EMIRGE (in purple) (Miller *et al.*, Genome Biology 2011, 12:R44), the RBG-2 and RBG-9 sequences (in red), previously sequenced genomes (in green), and a reference set mined from the SILVA database to encompass all currently known Chloroflexi clades. The alignment was generated using the SILVA SINA alignment algorithm with common gaps removed (Pruesse *et al.*, Nucl. Acids Res. 2007, 35:7188–96), resulting in a final alignment of 630 sequences and 1,094 unambiguously aligned positions. The phylogeny was conducted using PhyML under the HKY-85 model of evolution with 100 bootstrap resamplings. Bootstrap support values greater than 50 are displayed.

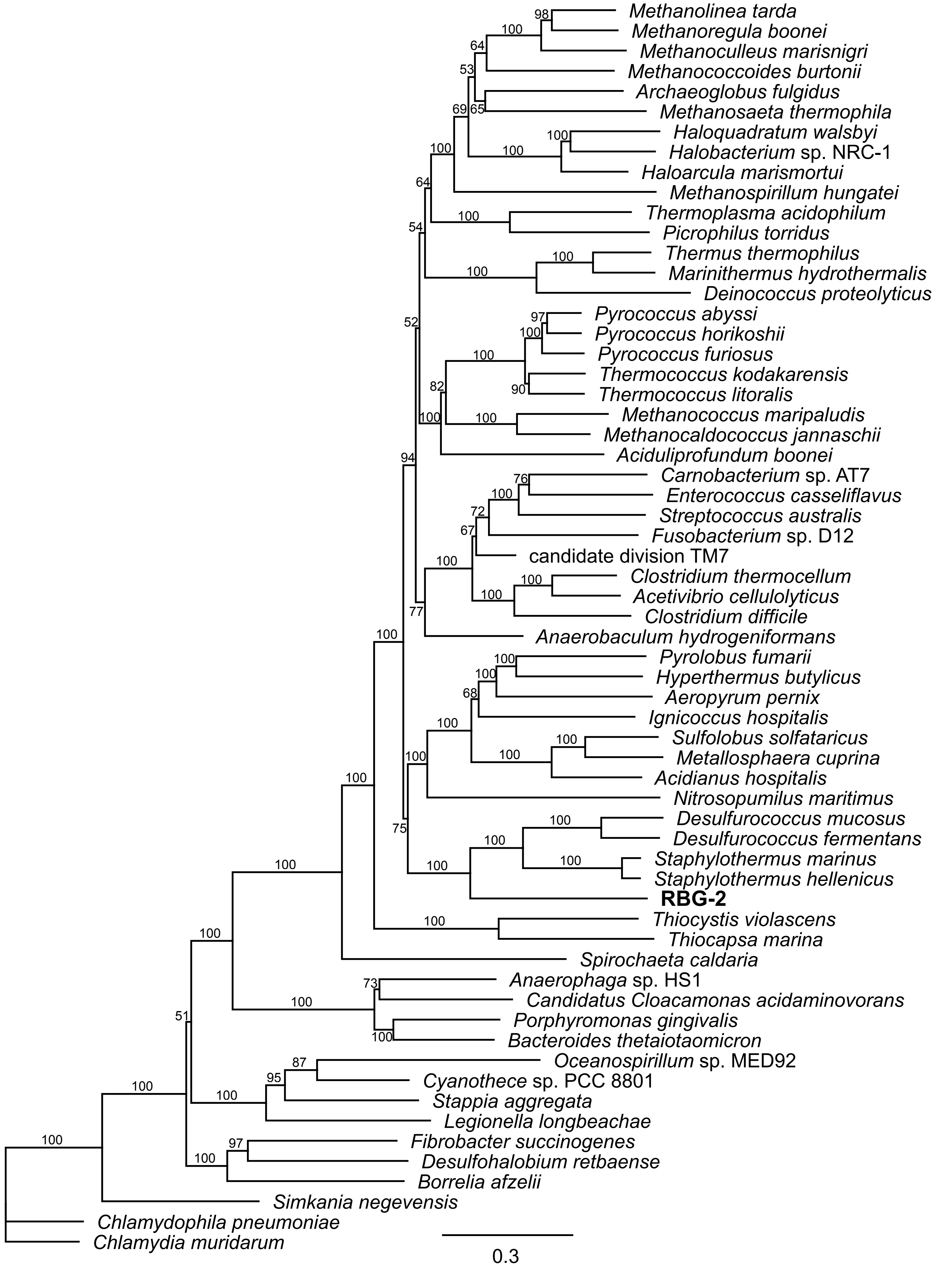

Figure S4: Concatenated maximum likelihood tree of Archaeal ATP-synthase subunits A, B, D, and I. For each subunit, sequences homologous to the RBG-2 proteins were mined from NCBI, and alignments were generated using Muscle v. 3.8.31 and manually curated. The alignments were concatenated into a final alignment of 62 taxa and 2,956 positions and the tree conducted using PhyML under the WAG+γ model of evolution. The position of the RBG-2 proteins was congruent in each of the single protein trees, but is better supported in the concatenated tree.

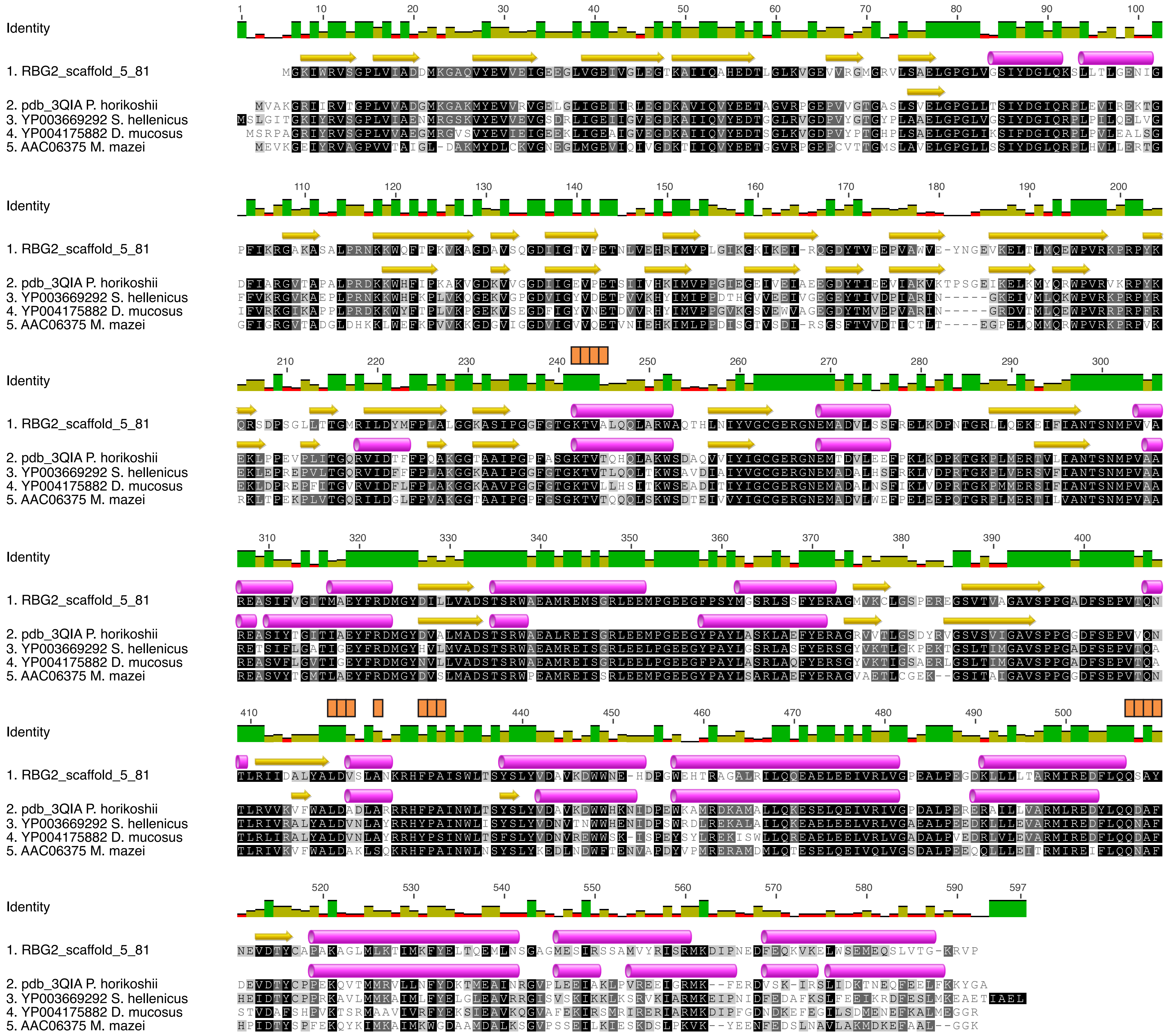

Figure S5: Alignment of A_1A_0-ATPase subunit A from RBG-2, the closest structural match (PDB entry 3QIA from *Pyrococcus horikoshii*), the two top BLASTp matches (*Staphylothermus hellenicus* DSM 12710 (YP_003669292) and *Desulfurococcus mucosus* DSM 2162 (YP_004175882)), and from *Methanosarcina mazei* Go1. The alignment is colored by similarity and identity is plotted as a histogram across the top. Structural elements are labeled in yellow for beta-sheets and pink for alpha helices. Structure annotations for P. horikoshii are from the determined crystal structure, while predictions for RBG-2 were derived from YASPIN and psipred. ADP-binding residues are marked with orange boxes.

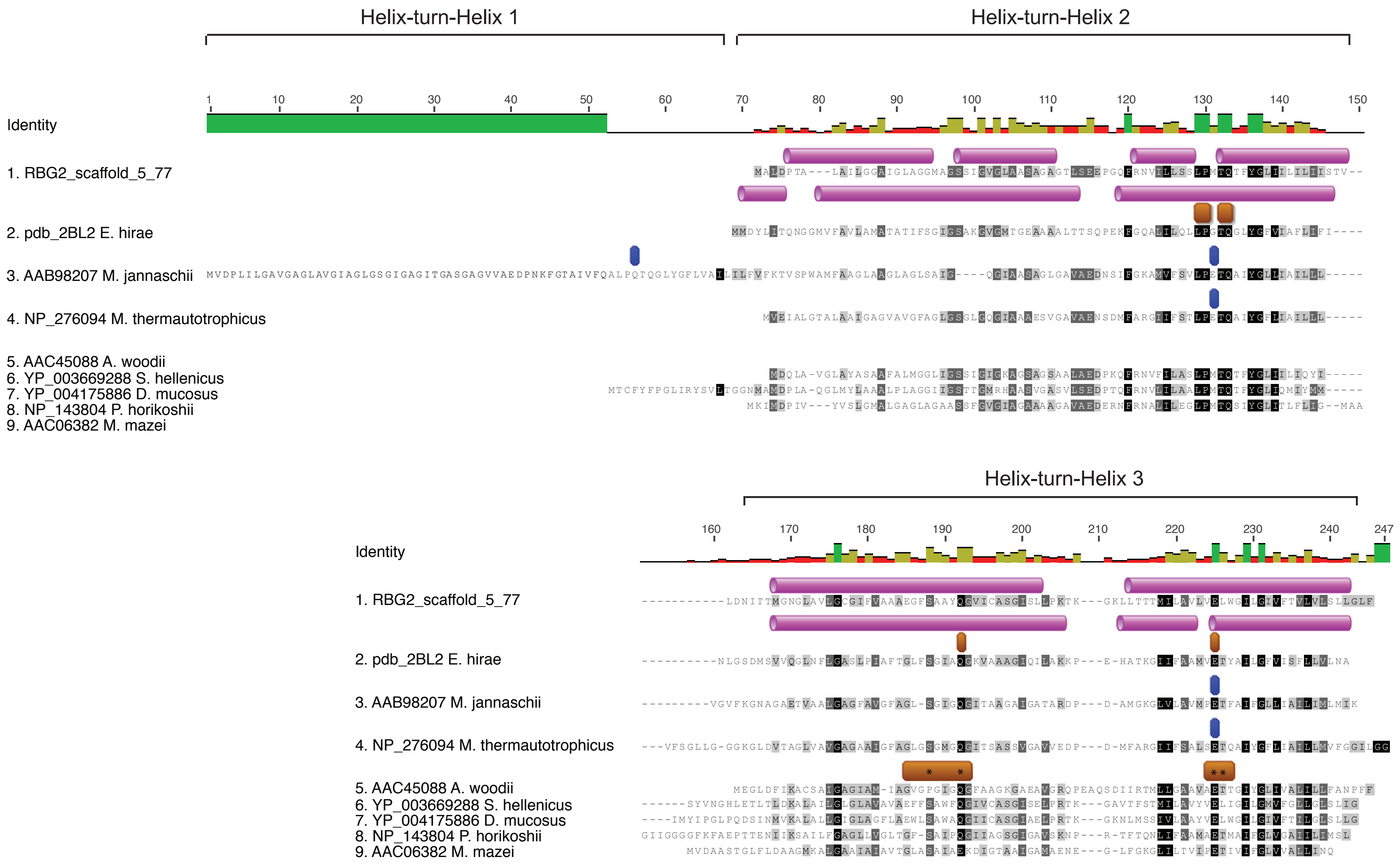

Figure S6: Alignment of putative A_1A_0-ATPase subunit K from RBG-2, the closest structural match (PDB entry 2BL2 from *Enterococcus hirae* GN), the top two BLASTp matches (*Staphylothermus hellenicus* DSM 12710 (YP_003669288) and *Desulfurococcus mucosus* DSM 2162 (YP_004175886)), H^+-translocating complex subunits from *Methanosarcina mazei* Go1 (AAC06382), *Methanocaldococcus jannaschii* DSM 2661 (AAB98207), and *Methanothermobacter thermautotrophicus* str. Delta H (NP_276094), the V-type K subunit from *Pyrococcus horikoshii* OT3 (NP_143804), and the Na^+-translocating complex subunit from *Acetobacterium woodii* DSM 1030 (AAC45088). The alignment is colored by similarity and identity is plotted as a histogram across the top. Structural elements are labeled in yellow for beta-sheets and pink for alpha helices. Structure annotations for *E. hirae* are from the determined crystal structure, while predictions for RBG-2 were derived from psipred. Critical carboxylates for H+ translocation are marked with blue boxes while Na+-binding residues are marked with brown boxes (asterisks denote key residues within the motif).

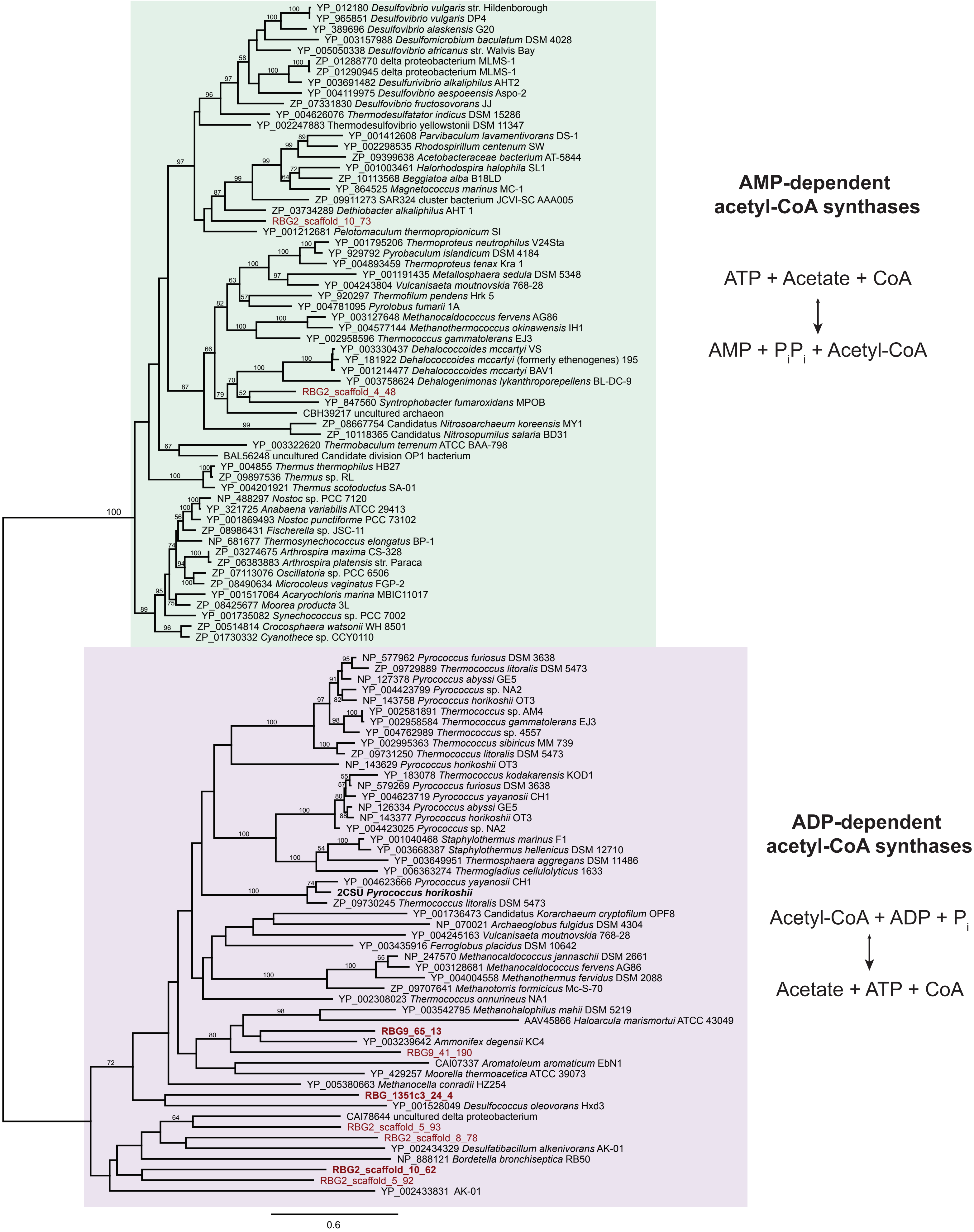

Figure S7: Maximum likelihood tree of ATP- and ADP-dependent acetyl-CoA synthetase alpha subunits, including putative enzymes from the three Chloroflexi genomes. Sequences in bold red correspond to alpha subunits affiliated with a beta subunit, forming a complete operon for ATP synthesis during the conversion of acetyl-CoA to acetate.

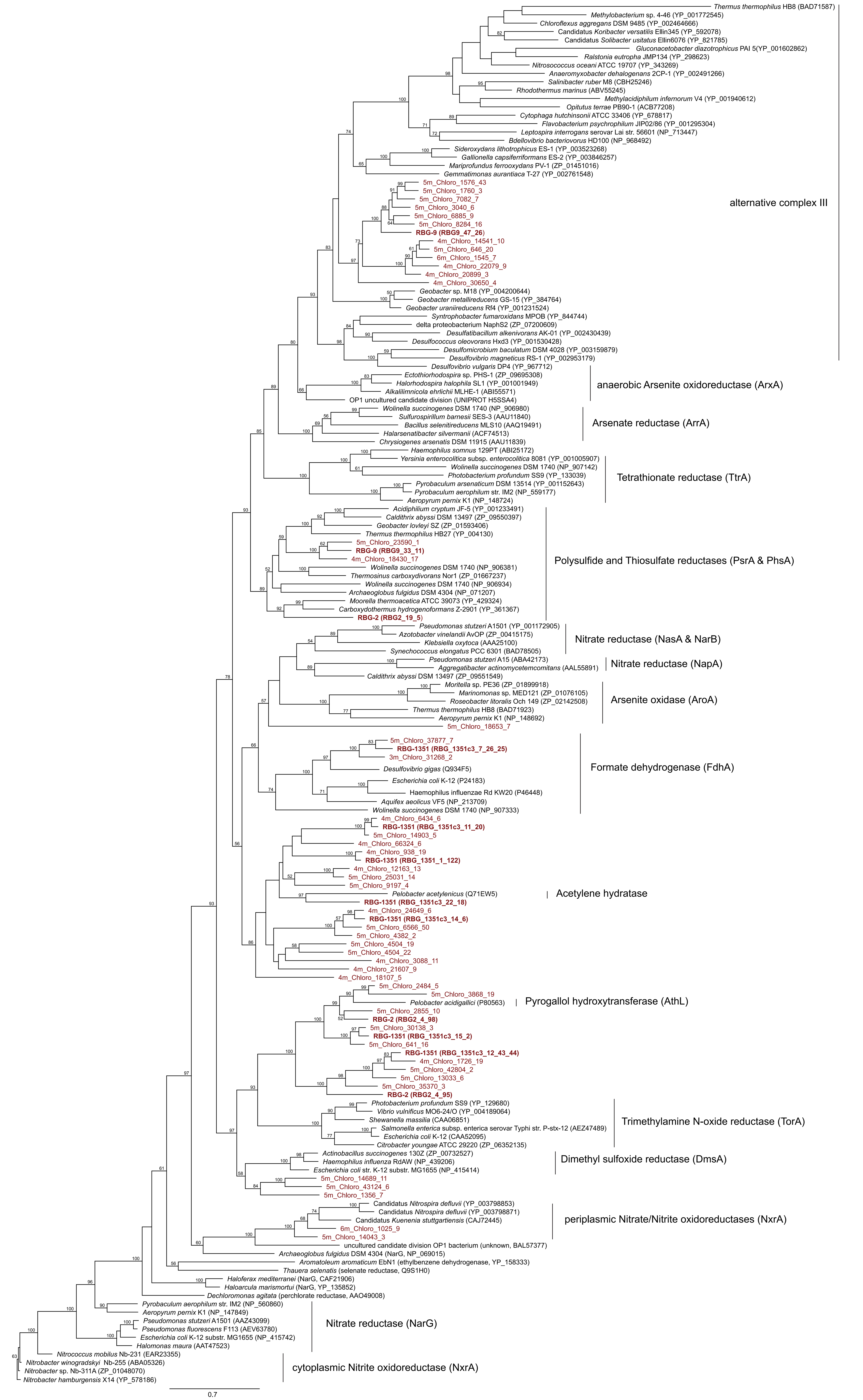

Figure S8: Maximum likelihood tree of catalytic subunits from the dimethyl-sulfoxide (DMSO) reductase superfamily. The tree contains all relevant characterized DMSO reductases, as well as the identified DMSO reductases from the Chloroflexi scaffolds and curated genomes. Chloroflexi sequences from this study are highlighted in red, and major clades of the superfamily are labeled. The tree is based on a protein alignment of 166 DMSO reductase sequences with 398 positions. The tree was conducted using PhyML under the WAG+γ model of evolution, with 100 bootstrap resamplings. Bootstrap support values greater than 50 are displayed.

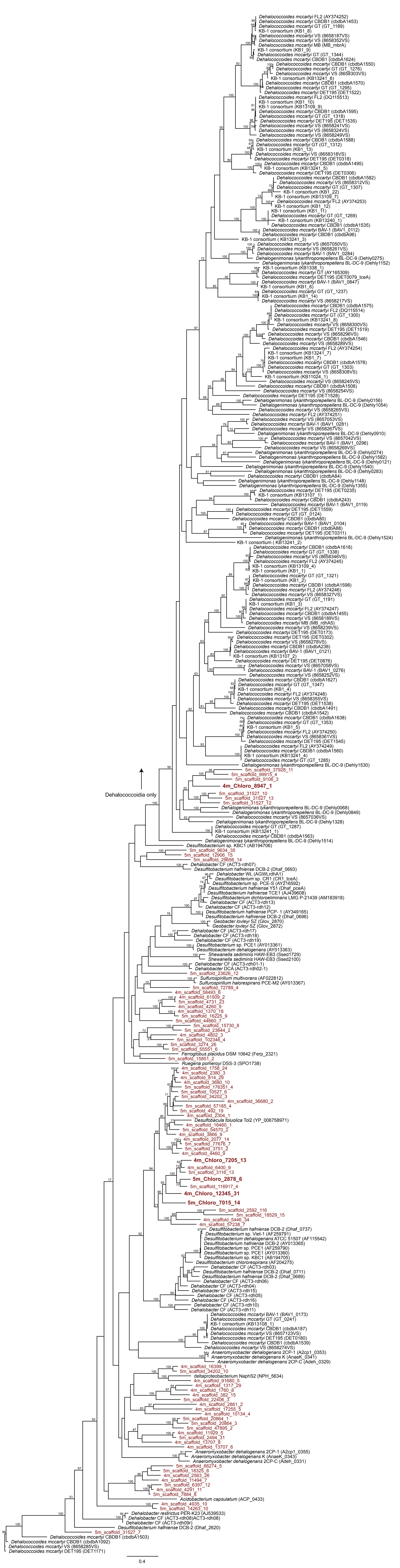

Figure S9: Maximum likelihood tree of reductive dehalogenase homolog catalytic subunits (RdhA). The tree contains all putative RdhA from the Rifle background metagenomes, as well as five RdhA encoded on the Chloroflexi scaffolds. No RdhA were identified from the three curated genomes. Rifle sequences are highlighted in red, with Chloroflexi sequences in bold. The tree is based on a protein alignment of 325 RdhAs with 315 positions. The tree was conducted using PhyML under the WAG+γ model of evolution, with 100 bootstrap resamplings. Bootstrap support values greater than 50 are displayed.
