# Supplementary material for: Community genomic analyses constrain the distribution of metabolic traits across the Chloroflexi phylum and indicate roles in sediment carbon cycling
Source: Microbiome. 2013 Aug 5;1:22. doi: 10.1186/2049-2618-1-22 (PMC3971608; doi:10.1186/2049-2618-1-22)
Supplement: Additional file 2 — Supplemental text. Supplemental notes on functions discussed in the main text as well as further information on the three Chloroflexi draft genomes (for example, oxygen tolerance, amino acid biosynthesis, and mobile element signatures). [file 2049-2618-1-22-S2.pdf]

Community genomic analyses constrain the distribution of metabolic traits across the Chloroflexi phylum and indicate roles in sediment carbon cycling

Laura A. Hug, Cindy J. Castelle, Kelly C. Wrighton, Brian C. Thomas, Itai Sharon, Kyle R. Frischkorn, Kenneth H. Williams, Susannah G. Tringe, and Jillian F. Banfield

Supplemental Information

Contents:

Supplemental notes on functions discussed in the main text.
- Hydrogenases
- Plant compound degradation (e.g., pyrogallol)
- Acetogenesis
- Sulfur and nitrogen
- RBG-2 supplementary notes
- RBG-1351 supplementary notes
- RBG-9 supplementary notes

Additional functions
- Arsenic resistance
- Oxygen tolerance
- Amino acid biosynthesis
- Fatty acid and nucleotide synthesis
- Co-factor biosynthesis
- Gene regulation and membrane transport
- Mobile elements and phage signatures
- Selenocysteine incorporation

Hydrogenases

The hydrogenases on the RBG-2 genome likely perform different functions. The Hup and Mbh hydrogenases are predicted to localize to the cytosolic side of the cell membrane, while the Mvh hydrogenase is predicted to be cytosolic. The dimeric hydrogen uptake hydrogenase (HupLS) belongs to group II of the Ni,Fe-hydrogenases [1], and is most closely related to Dehalococcoidia Hup proteins. Its primary function is likely the oxidation of H_2 with concomitant transfer of H^+ across the membrane. The trimeric F-420 non-reducing-like H_2ase (MvhADG) belongs to group III of the Ni,Fe- hydrogenases [1], and is most closely related to Firmicute hydrogenases. The Mvh hydrogenases have

been shown to provide reducing equivalents to heterodisulfide reductase (Hdr) during methanogenesis in Archaea [2, 3]. RBG-2 encodes a heterodisulfide reductase enzyme complex with highest sequence similarity to Deltaproteobacterial HdrABC, but does not encode the required genes for methanogenesis or coenzyme M biosynthesis. While an interaction between the Mvh hydrogenase and the heterodisulfide reductase is hypothesized, the overall function and substrates of the complex is not clear. The third hydrogenase is a membrane-bound hydrogenase of Archaeal origin, comprising 14 subunits encoded as a gene cluster (Mbh subunits A-N). The Mbh hydrogenases contain four subunits homologous to Complex I, and are able to couple oxidation of a carbonyl group with reduction of protons to H_2 [1, 4]. In *Pyrococcus furiosus*, the Mbh couples electron transfer from reduced ferredoxin to both proton translocation and H_2 evolution [5]. In RBG-2 the electron donor to Mbh is likely also reduced ferredoxin, generated by PFOR and the other ferredoxin oxidoreductases. The co-presence of the H_2-evolving Mbh with H_2-oxidizing Hup and Mvh hydrogenases presents the possibility of intra-species hydrogen transfer between hydrogenase complexes, where H_2 evolved by Mbh is reoxidized by either of the other two H_2ases [6].

The RBG-1351 Ni,Fe hydrogenase is most similar to the complex in *Dehalogenimonas lykanthroporepellens* BL-DC-9. The RBG-1351 genome additionally contains the HymA, B, and C2 subunits for a *Dehalococcoides*-related Fe-only hydrogenase, but does not contain a homolog to the HymC catalytic subunit, nor are any Fe-hydrogenase maturation protein genes present. The operon is located near a partial recombinase gene where the large C subunit was expected, indicating genome rearrangements may have resulted in loss of this subunit and hence this activity.

Ferredoxins are prevalent in both the annotated near-complete genomes and in the larger Chloroflexi metabolic potential. RBG-2's genome encodes nine ferredoxin genes, RBG-1351's sixteen, and RBG-9's thirteen. None of the curated genomes encode genes for quinone synthesis, indicating ferredoxins are likely the primary electron shuttle. In the larger Chloroflexi sequence dataset, the number of ferredoxins was approximately six times the number of predicted organisms for all three depths, further support for ferredoxins being a primary electron shuttle within the Chloroflexi.

Plant compound degradation

Pyrogallol is an important component of plant polymers. The pathway from pyrogallol to acetate requires an NADPH-dependent phloroglucinol reductase [7], followed by several as-yet undefined hydrolysis and other reactions, yielding (S)-3-hydroxy-butanoyl-CoA, which is converted to acetoacetyl-CoA by 3-hydroxybutyryl-CoA dehydrogenase and then to acetyl-CoA by acetyl-CoA C-acetyltransferase. The RBG-2 pyrogallol transhydroxylases have highest sequence similarity to a putative pyrogallol transhydroxylase from the Firmicute *Desulfitobacterium hafniense* st. DBC-2 [8]. RBG-2 encodes 3-hydroxybutyryl-CoA dehydrogenase as well as acetyl-CoA C-acetyltransferase, the only other described portions of pyrogallol fermentation. The two RBG-1351 pyrogallol transhydroxylases have highest sequence similarity to genes from *Desulfitobacterium* and *Holophaga*. All four pyrogallol transhydroxylases are predicted to be cytoplasmic by psortB (version 3.0.2, [9]). Modeling confirms the presence of shared structure with crystallized pyrogallol transhydroxylases.

The two predicted endoglucanases on the RBG-9 genome belong to the M42 family, containing aminopeptidases, cellulases, and endoglucanases. They bear highest sequence similarity to genes from Firmicutes and *Anaerolinea thermophila* UN-1, respectively. The beta-glucosidase gene has highest sequence similarity to a gene annotated as a "broad-specificity cellobiase" from *Gloeocapsa*.

Acetogenesis

RBG-2 encodes four predicted genes for ADP-dependent acetyl-CoA synthetase I (ACS-I) [10], three missing a downstream beta subunit. The fourth is a complete operon of alpha and beta subunits with structural similarity to the ACS-I from *Pyrococcus horikoshii* OT3 (pdb model: 2csu) (Figure S7). The RBG-1351 genome contains a single ACS-I operon of alpha and beta subunits with structural similarity to the ACS-I from *Pyrococcus horikoshii* OT3 (pdb model: 2csu) and highest sequence similarity with ACS-I from *Pyrococcus yayanosii* CH1 (Figure S7). RBG-9 contains two copies of ACS-I for formation of acetate and ATP from acetyl-CoA and ADP.

Sulfur and Nitrogen

RBG-2 encodes two aryl sulfatases, enzymes that hydrolyze sulfate esters to sulfate, and which are implicated in sulfur scavenging from the environment. Arylsulfatases are typically extracellular, and associated with mineralization of sulfur for plant utilization. The Arylsulfatase encoded by RBG-2 is predicted to localize to the cytosol [9], suggesting it is not directly interacting with the sediment environment. RBG-1351's genome contains one putative sulfane dehydrogenase, which oxidizes protein-bound sulfide. RBG-9's genome encodes a single sulfotransferase and two DsrE proteins for oxidation of intracellular sulfur. Sulfate and sulfite conversions were not strongly represented in the general Chloroflexi dataset: only non-catalytic subunits of dissimilatory sulfite reductase (dsrC) and sulfite oxidase (YedF) were identified. Heterodisulfide reductases were more common, though full HdrABC operons are present well below the predicted organism numbers.

RBG-2 and RBG-1351's genomes each encode 3 nitroreductases and two putative nitroreductases (type III). The nitroreductases are predicted to catalyze the reduction of nitroaromatic compounds, including nitrotoluenes. The two type III nitroreductases belong to a group of uncharacterized enzymes whose substrates are unknown. The blue-copper containing nitrite reductase on the RBG-9 genome shares highest sequence similarity with a gene from *Nitratireductor pacificus* pht-3B, an Alphaproteobacteria. Aside from the blue-copper nitrite reductase, the RBG-9 genome contains three putative nitroreductases: two associated with the type III nitroreductases and one with the NAD(P)H:flavin oxidoreductase-like family 1 (substrate unknown).

RBG-2 metabolism supplementary notes

In the RBG-2 genome the oxidative upper portion of the pentose phosphate pathway was not identified, but transaldolase, transketolase, and glucose phosphate isomerase predicted proteins are present on the genome, indicating formation of 5-C sugars is possible, but is uncoupled from NADPH formation.

The RBG-2 ATP synthase is encoded as a nine-gene operon, *ahaHIKECFABD*. The operon structure is most similar to A_1A_0-type ATPases, which typically contain nine to ten genes and catalyze the formation of ATP, leveraging a proton motive force across the cell membrane. In contrast, the homologous V_1V_0-type ATPases typically contain 13 or more subunits [11], and catalyze only the reverse reaction: hydrolyzation of ATP for formation of an ion gradient. The RBG-2 ATP synthase lacks only the G subunit, a gene putatively associated with characterized A_1A_0-ATP synthases. The G subunit is absent in several A_1A_0-ATP synthase operons and in characterized protein complexes [11]. The ATP synthase proteolipid, subunit K, allows inference of the catalytic activity of the complex. Typical A_1A_0 proteolipids are 8 kDa proteins with a helix-turn-helix (HTH) structure, with a conserved glutamate in the second helix required for proton translocation [11]. The RBG-2 predicted proteolipid is a ~16 kDa protein composed of two helix-turn-helix motifs, a duplicated structure similar to that seen in V_1V_0 ATPase proteolipids, *Methanothermobacter thermautotrophicus* str. Delta H A_1A_0-ATP synthase, and in Crenarchaeotal complexes [11] (Figure S6). The RBG-2 sequence contains the conserved glutamate in the second HTH only (Figure S6), with a substituted methionine in the first HTH. Activity studies on ATP synthases have shown a requirement for translocation of 3-4 H^+ for concurrent ATP synthesis to be energetically feasible, with a current observed minimum of 2.6 H^+ per ATP. With a four-transmembrane-helix proteolipid and only one conserved carboxyl, RBG-2's ATP synthase is predicted to translocate only 2 H^+ per revolution of the membrane-bound complex. However, the structure and stoichiometry of Crenarchaeotal ATP synthases have not been determined, and it is difficult to predict the interplay of the proteolipid subunits with confidence in the absence of redox information. As a final note, the *Acetobacterium woodii* DSM 1030 ATP synthase is a Na^+-translocating complex. The Na^+-binding sites on the *A.* woodii proteolipid have been determined [12, 13], but are conserved in both RBG-2 and in known proton-translocating A and V-type ATP synthases (Figure S6), making the presence of these residues uninformative to this discussion.

The identified haloalkane dehalogenase in RBG-2 is structurally similar to DppA from the Deltaproteobacterium *Plesiocystis pacifica*, and has highest sequence similarity (54%) to a putative haloalkane dehalogenase in the Chloroflexi *Ktedonobacter racemifer*

DSM 44963. The protein sequences for DppA and the RBG-2 putative haloalkane dehalogenase share 39% identity, including conservation of the catalytic triad and the chloride-binding pocket residues. DppA is active on bromo-alkanes, especially bromobutane [14], though substrate specificity cannot be inferred from this for the RBG-2 haloalkane dehalogenase. Both *Ktedonobacter* and *Plesiocystis* are strictly aerobic organisms, and haloalkane dehalogenases are aerobic enzymes, making the presence of a haloalkane dehalogenase in RBG-2 unexpected.

RBG-1351 metabolism supplementary notes

The ability to ferment glucose to propionate may provide RBG-1351 a mechanism to utilize succinyl CoA that cannot be converted to succinate due to the incomplete TCA cycle. The genome contains a complete fermentation pathway from succinyl-CoA to propionate, making succinyl-CoA synthetase the only missing enzyme for fermentation of glucose through to propionate. A 3:oxoacid CoA-transferase (E.C. 2.8.3.5) is present, and may catalyze the conversion of succinate to succinyl-CoA. The RBG-1351 propionate fermentation pathway relies on two assumptions. 1: That the 3:oxoacid CoA-transferase is active on succinate. It is additionally possible the succinyl-CoA synthetase alpha and beta subunit genes are missing from the draft genome but present in the complete RBG-1351 gene complement. 2: That the propionyl-CoA carboxylase reaction is reversible, forming propionyl-CoA from (S)-methylmalonyl-CoA. This reaction has been shown to be reversible under low propionyl-CoA flux in mammals [15], but its reversibility (or lack thereof) in bacteria is not documented.

The RBG-1351 genome encodes three oxidoreductases closely related to the tungsten iron-sulfur enzyme acetylene hydratase from mesophilic *Pelobacter acetylenicus*, which catalyzes acetylene to acetaldehyde acetaldehyde (Figure S8). The structure of the enzyme from *Pelobacter acetylenicus* has been resolved giving more insights about the active site and the substrate-binding motif [16, 17]. Amino acid sequence analysis reveals the presence of the [4Fe-4S] cluster for three of the enzymes from RBG-1351, however a critical catalytic residue, an aspartate in position 13 is missing in all three predicted proteins. Moreover, one hydrophobic residue, an Ile in position 142 that forms part of the

substrate cavity and thus is key determinant for the specificity of the substrate [17], is also missing. Finally a Lys in position 48 is also absent in two of the three RBG-1351 proteins. The absence of these critical residues suggests that the enzymes identified in RBG-1351 may act on a substrate other than acetylene, and thus may represent a new kind of oxidoreductase in the DMSO reductase family.

The RBG-1351 genome contains a partial bacterial F_0F_1-ATP synthase operon (F_0 ABC, F_1 $\alpha\delta$) at the end of a scaffold. F_1 subunits $\beta\gamma\varepsilon$ are likely present, but missing from the draft genome.

RBG-9 supplementary notes

NADH dehydrogenase complex I genes are duplicated in the RBG-9 genome. A single operon containing the *nuoABC/DEFGHIJK* subunits represents a near-complete complex. Separate from this operon are operons containing *nuoLM*, *nuoBC/DI*, and *nuoAHJKLMN*. All of the predicted complex I genes are most closely related to Chloroflexi genes. RBG-9 also encodes two operons for complex II (succinate dehydrogenase/fumarate reductase), one most closely related to Chloroflexi complexes, and one with highest similarity to Firmicute genes.

The RBG-9 genome contains putative electron transport chain genes belonging to the superfamily of DMSO reductases. The members of the DMSO family are generally composed of three subunits: the catalytic subunit (a molybdenum containing cofactor and in most cases a [4Fe-4S] cluster), the iron sulfur containing subunit (either four [4Fe-4S] or three [4Fe-4S] and one [3Fe-4S] clusters) and an integral membrane protein. Phylogenetic analysis of the alternate complex III (ACIII) catalytic subunit reveals it is closely related to molybdopterin oxidoreductases identified in iron oxidizers such as *Mariprofondus ferrooxydans* [18] and iron reducers such as *Geobacter metallireducens*. ACIII, a membrane-bound complex widespread in the bacterial domain [19], functionally replaces the bc_1 complex (complex III) [20, 21]. The gene cluster encoding for ACIII may contain up to eight open reading frames encoding diverse subunits (ActA,B,C,D,E,F,G) [22, 23]. ActB is a catalytic subunit resulting from the fusion of two domains designated B1 and B2, homologous to two subunits of other DMSO reductase

enzymes (the catalytic subunit and the iron sulfur containing subunit, respectively). In some organisms, two distinct genes encode these domains. Investigation of the genomic context of the RBG-9 ACIII reveals a very similar organization of genes encoding ACIII: ActA, B1, B2, C, D, E. Subunits ActF and ActG are missing. The same composition and organization of the cluster is present in the Deltaproteobacterium *G. metallireducens* [23]. Singer et al. suggested that the genes encoding for ACIII from *M. ferrooxydans* might play a role in electron transport during iron oxidation [18]. The genes encoding ACIII are often clustered with those for an oxygen reductase (Complex IV; [19]), and form a functional association with the caa_3-type cytochrome *c* oxidase in *R. marinus* [24]. In RBG9, the gene cluster does not include an oxygen reductase, but a caa_3-type cytochrome *c* oxidase is present in the genome, suggesting the ACIII might participate in electron transfer coupled to oxygen reduction.

The thirty-five uncharacterized oxidoreductases in the RBG-9 genome have putative annotations including FAD-dependent, FeS-containing, FMN-linked, coenzyme F420-dependent, and the more general "putative oxidoreductase". The precise mechanism of these oxidoreductases is not clear, but indicates substantial capacity for electron transfer.

Genes for utilization of mannose, fuculose, rhamnose, lactose, tagatose, and alginate were not identified. Several genes involved in aromatic compound degradation, including a complete 3-methylbenzoyl-CoA reductase complex and a 6-oxo-cyclohex-1-ene-carbonyl-CoA hydrolase are present, but the capacity to derive energy from aromatic ring compounds is not present.

Lysine and glutamate fermentation may be catalyzed by glutamate dehydrogenase, while glutamate may be alternatively metabolized using glutamate synthase to form glutamine and 2-oxoglutarate, which can enter the TCA cycle as an intermediate. Glutamate dehydrogenase may alternatively work in ammonia assimilation, forming glutamate. Alanine and aspartate can be fermented to pyruvate through the actions of a fumarase, a unidirectional aspartate-ammonia lyase, and malic enzyme. A partial phenylacetic acid pathway is present, but lacks several key enzymes for full degradation of phenylalanine.

The RBG-9 genome contains several unusual features compared to RBG-2 and RBG-1351, including two genes associated with plasmid maintenance systems. This indicates the RBG-9 genome may exist as multiple chromosomes, a structure seen in other Chloroflexi genomes to date [25–27]. The RBG-9 genome also contains a partial Eut operon for synthesis of an ethanolamine degradation carboxysome, a protein-encased microcompartment for sequestration of specific cellular activities [28]. The operon on the genome contains major structural components of a carboxysome: homologs to *eutM* and *pduT*, which form hexahedral structural units, and *eutN*, which forms pentahedral structural units. The operon also contains a homolog to *eutE*, a Coenzyme A acylating aldehyde dehydrogenase responsible for conversion of acetaldehyde to coenzyme A. Missing from this operon are *eutB* and *eutC*, the ethanolamine ammonia lyase large and small subunits, required for utilization of ethanolamine. Also missing are many of the typical accessory structural proteins (*eutSPQLK)*.

Additional functions

Arsenic resistance

Arsenate (As(V)) can enter a cell via phosphate transporters, and competitively binds in place of phosphate, disrupting core cellular processes [29]. The Rifle IFRC sediment contains arsenic at approximately 1.5 µM, which increases during acetate amendment [30], meaning the presence of arsenic resistance systems may be of particular importance for the environment these Chloroflexi inhabit. RBG-2 encodes a complete arsenic resistance pathway, including Arsenate reductase (*arsC*) for conversion of arsenate to arsenite (As(III)), arsenite permease, an arsenite transporter, and three transcriptional regulators (*arsR*). This system can be used to excrete invading arsenate, protecting the cell, but lacks the genes associated with energy derivation from this process [29]. RBG-1351 contains a different mechanism of arsenic resistance in the form of a gene encoding an arsenite S-adenosylmethionine methyltransferase (ArsM), which methylates and volatilizes arsenite [31]. RBG-1351 also encodes an ArsR transcriptional regulator, though it is not in an operon with the ArsM. RBG-1351 may alternatively excrete arsenite, utilizing arsenite permease, an arsenical pump protein, and an arsenite

transporter. The RBG-9 genome encodes eleven *arsR* transcriptional regulator genes, along with an arsenite transporter and *arsC*, an arsenate reductase. RBG-9 does not encode an arsenite permease homolog.

Oxygen tolerance

Oxygen and free radical scavenging in RBG-2 is encoded by a Fe-Mn family superoxide dismutase, a superoxide reductase, a peroxidase/catalase, and a glycolate oxidase. Also present are a rubredoxin and four predicted rubrerythrins, genes putatively involved in oxygen scavenging [32, 33]. RBG-2 also encodes a Type IV RuBisCO, a subfamily of non-carbon-fixing RuBisCOs [34]. The RBG-2 RuBisCO is a YkrW-type Type IV RuBisCO, a class of enzymes implicated in the response to oxidative stress [35] as well as methionine salvage [34] and sulfur metabolism. RBG-1351 shares a similar oxygen tolerance profile to RBG-2, encoding a type III catalase, Fe-Mn family superoxide dismutase, a rubredoxin, and three rubrerythrin genes, but no type IV RuBisCO. The presence of SOD and peroxidase/catalase indicates RBG-2 and RBG-1351 are possibly oxygen tolerant, though the absence of oxygen reductases indicates anaerobic metabolism. The RBG-9 genome contains a single Fe-Mn superoxide dismutase alongside two peroxiredoxins, three alkyl hydroperoxide reductases, and one acid phosphatase/vanadium-dependent haloperoxidase-like protein for hydrogen peroxide scavenging. Given the presence of cytochrome *c* oxidase as a terminal oxygen acceptor, it is likely RBG-9 is aerobic, or microaerophilic.

Amino acid synthesis

RBG-2 encodes biosynthesis pathways for 19 of the 20 amino acids. The aromatic-containing amino acids are likely synthesized using a bifunctional 3-dehydroquinate synthase/shikimate kinase. Arginine formation from aspartate within the urea cycle is encoded, but an arginase is missing, making the urea cycle incomplete. Biosynthesis of alanine is encoded by cysteine desulfurase (alanine biosynthesis III). The methionine synthesis and salvage pathways are incomplete. The methionine synthesis and salvage pathways are also patchily represented in the *Dehalococcoides* [36–38], though *Dehalococcoides mccartyi* strains are not methionine auxotrophs [39], indicating either

all required enzymes are present and currently unidentified, or a novel process is occurring. From this, it is possible RBG-2 is not a methionine auxotroph despite the seeming absence of a synthesis pathway.

RBG-1351 appears to be auxotrophic for nearly all amino acids. Complete biosynthesis pathways were identified for histidine, cysteine (from pyruvate) and phenylalanine only. Synthesis of alanine, glutamine, glutamate, asparagine, aspartate, threonine, serine, tyrosine, lysine, isoleucine, valine, leucine, and methionine is not encoded on the genome. Arginine, proline, tryptophan, and glycine can be generated from serine, aspartate, and/or glutamate, but biosynthesis of those precursor amino acids is not encoded. The methionine salvage pathway is likewise very incomplete.

RBG-9 falls between RBG-2 and RBG-1351 in terms of the number of amino acids it is predicted to synthesize. Complete synthesis of leucine, isoleucine, valine, proline, alanine, glycine, serine, threonine, and arginine is encoded on the genome. Glutamate can be synthesized from carbamate or proline, while glutamine synthesis is incomplete. Incomplete synthesis pathways are present for histidine, cysteine, aspartate and asparagine, and methionine. Like the other two genomes, the methionine salvage pathway is incomplete.

Fatty acid and nucleotide synthesis

RBG-2 encodes enzymes for saturated fatty acid synthesis from acetyl-CoA and malonyl-CoA through to enoyl-[acyl carrier proteins]. RBG-2 lacks any predicted enzymes involved in acyl-chain termination: thioesterases A and B, oleoyl-[acyl-carrier-protein] hydrolase, and fatty acid synthase are all missing from the genome. The glycerophospholipid pathway is complete from glyceraldehyde-3P to glycerone phosphate, and subsequently to phosphatidyl-glycerophosphate. Contrastingly, the glycerolipid synthesis pathway from glycerate is incomplete, ending at 1,2,-Diacyl-sn-glycerol 3-phosphate. Saturated and unsaturated fatty acid biosynthesis is not complete on the RBG-1351 genome. Genes for FabD, FabF, and FabG are present, but aside from a predicted FabK, the lower half of the saturated fatty acid synthesis pathway (FabAZI) is not present. Glycerolipid and glycerophospholipid biosynthesis is similarly incomplete.

Mechanisms for synthesis and subsequent conversion of glycerol to glycerol-3-phosphate are missing, as are genes for formation of fatty acids or triacylglycerol from any diacyl-glycerol derivatives. The glycerophospholipid pathway is partially present, and is predicted to function in the conversion of glycerone phosphate and glycerol-3-phosphate to phosphatidyl-glycerophosphate as in RBG-2. However, the absence of genes for formation of either glycerone phosphate or glycerol-3-phosphate makes the function of this pathway uncertain in RBG-1351. Fatty acid synthesis is near complete on the RBG-9 genome. Similar to the RBG-2 genome, the final steps for acyl-chain termination are absent. The glycerophospholipid pathway is complete for synthesis of phosphatidyl-glycerol, 1,2,-Diacyl-glycerol, and phosphatidyl-ethanolamine from glycerone phosphate. Glycerolipid synthesis is incomplete.

RBG-2 encodes complete biosynthesis pathways for purine and pyrimidine nucleic acids, as well as DNA and RNA synthesis from those precursors. Complete synthesis pathways for pyrimidine and purine nucleotides are encoded on the RBG-1351 genome, as are all genes expected for RNA synthesis. Conspicuously absent is a homolog for *nrdD*, the ribonucleoside-triphosphate reductase required for conversion of nucleotides to deoxyribonucleotides for DNA synthesis. Given the obligate nature of this function, this appears to be a result of genome incompleteness rather than a true gene absence. RBG-9's genome contains complete biosynthesis pathways for purine and pyrimidine nucleic acids, as well as DNA and RNA synthesis from those precursors. Unlike the RBG-2 and RBG-1351 genomes, the RBG-9 genome contains the genes for synthesis of xanthine and inosine precursors within purine formation.

Cofactor biosynthesis

All three curated genomes lack a complete corrinoid synthesis pathway, but do encode corrinoid-cofactor-containing proteins. Similar to the *Dehalococodides*, RBG-1351 encodes the lower portion of the pathway (cobQ, cobC, cobU, cobS, cobT), allowing conversion of corrin-containing molecules to the correct compound. RBG-2 and RBG-9 do not have this capacity. Each of the genomes contains a cobalamin/Fe^{3+} siderophore ABC transporter for import of corrinoids.

RBG-2 and RBG-1351 encode full and near-complete MEP/DOXP pathways for synthesis of geranylgeranyl pyrophosphate and genes for its conversion to menaquinone or phyloquinone. RBG-1351 is missing farnesyl diphosphate synthase, geranylgeranyl diphosphate synthase, and geranylgeranyl reductase. Unlike RBG-2 and RBG-1351, the RBG-9 genome contains a partial mevalonate pathway for geranyl-PP synthesis, as compared to the MEP/DOXP pathway. The genome lacks phosphomevalonate kinase and farnesyl diphosphate synthase. Additionally, the RBG-9 genome does not contain genes for *de novo* synthesis of octaprenyl-pyrophosphate or phytyl-pyrophosphate from geranyl-PP, but does encode genes for their conversion to menaquinone and phylloquinone. In all three genomes, the ubiquinone synthesis pathway is incomplete or entirely absent: it is unlikely quinones are a major component of electron transport in these organisms.

All three genomes have complete biosynthesis pathways for molybdopterin and for pyridoxal 5-phosphate but not pyridoxine. The pantothenate and CoA synthesis pathway is complete in RBG-2 and RBG-1351. RBG-9 lacks a pantetheine hydrolase, but all other genes are present. RBG-2 encodes biosynthesis of riboflavin and FAD, as well as NAD and NADP from aspartate. RBG-1351 and RBG-9 can synthesize FAD from riboflavin, but likely cannot make riboflavin *de novo*. RBG-9 encodes a complete pathway for nicotinamide and nicotinate from NAD^+ and NADH, while RBG-1351's pathway is incomplete, missing L-aspartate oxidase and NAD^+ synthase. RBG-9 contains complete biosynthesis pathways for thiamine pyrophosphate, biotin, and coenzyme F420, pathways that are incomplete in RBG-2 and RBG-1351. None of the genomes contain complete synthesis pathways for heme, carotenoids, or glutathione.

RBG-2 and RBG-1351 have incomplete synthesis pathways for thiamine pyrophosphate (TPP), folate, dihydrofolate, and tetrahydrofolate. The absence of a complete tetrahydrofolate synthesis pathway is unexpected, however both genomes encode a large number of genes associated with the "one-carbon pool by folate" KEGG pathway, indicating folate that is present or scavenged can be converted to different, usable forms. In RBG-9, *de novo* synthesis of dihydrofolate and tetrahydrofolate is incomplete, missing dihydroneopterin aldolase and dihydrofolate reductase, however methyl-tetrahydrofolate

can be generated from phosphoenolpyruvate via the ribulose monophosphate pathway through formaldehyde formation. Folate conversions are more restricted than in RBG-2 and RBG-1351, limited to those reactions associated with amino acid synthesis.

Gene regulation and membrane transport

RBG-2, RBG-1351, and RBG-9 regulate gene expression and response to environmental changes with suites of 87, 77, and 217 genes respectively. These include histidine kinases within two-component regulation operons, and a variety of one-component regulators. The one-component regulators include GntR, MarR, PadR, ArsR, and others. For all three genomes, the targets for gene expression regulation or the input signals sensed by these proteins cannot be speculated from sequence data alone.

The RBG-2 genome contains genes for ABC transporters specific to tungstate, phosphate, antibiotics (specifically daunorubicin), branched-chain amino acids, oligopeptide/dipeptide/nickel, and iron complexes/cobalamin are present. Antibiotic resistance is additionally encoded by a glyoxylase/bleomycin resistance protein. Beyond the ABC transporter family, RBG-2 encodes membrane transporters for Mg^{2+}, Ca^{2+}, Na^{+}, chromate, citrate, ammonium, manganese/iron, cobalt, and arsenite. RBG-2 encodes a type II secretion pathway, but does not have a twin-arginine translocation system (TAT). This absence is reflected in the number of proteins with a TAT signal sequence: only four (0.2%) predicted open reading frames contain the TAT motif, while a further 174 contain a putative TAT signal sequence based on TATP [40], but are lacking the strict twin arginine motif (Table 1). This may be evidence for gradual loss of the TAT signals following the loss of the TAT pathway, which is present on other Chloroflexi genomes [36, 37].

The RBG-1351 genome contains ABC transporters for oligopeptides, dipeptides, branched chain amino acids, nickel, cobalamin and iron complexes, molybdenate, tungstate, phosphonate, and antibiotic resistance (daunorubicin specifically). Other transporters in the genome allow import and export of magnesium, cobalt, sodium, ferrous iron, copper, heavy metals, amino acids, polyamines, potassium, arsenite, and

phosphate. In addition to basic transport, the RBG-1351 genome contains complete TAT and Type II secretion systems for protein export or localization to the cell membrane.

The RBG-9 genome has a substantially larger repertoire of transporter genes compared to the RBG-1351 and RBG-2 genomes. The RBG-9 genome contains ABC transporter complexes for simple sugars, monosaccharides, arabinose, branched chain amino acids, polar amino acids, L-amino acids, dipeptides, peptides, lipoproteins, zinc/manganese, iron, cobalt/nickel, cobalamin, tungstate, potassium, phosphonate, urea, heme, molybdenate, cobalamin, antibiotics (macrolides, daunorubicin), nitrate, sulfonate/nitrate/nitrate/taurine and spermidine/putrescine. Other transporters are annotated for translocation of sugars, arabinose, ribose, amino acids, basic amino acids/polyamines, branched chain amino acids, neutral amino acids, lysine, peptide/nickel, copper, potassium, ferrous iron, Ca^{2+}, phosphate, nitrite, formate/nitrite, sulphate, sodium/sulphate, chromate, arsenite, heavy metals, spermidine/putrescine, fosmidomycin, and biopolymers. The RBG-9 genome contains complete TAT and Type II secretion systems for translocation of proteins across the cell membrane.

Mobile elements and phage signatures

RBG-2 encodes four putative transposases, one integron cassette protein, and thirteen integrases. RBG-2's transposases include one mutator-type, two belonging to the IS3/IS911 family, and one IS200 family. RBG-2 also encodes one integron cassette protein and thirteen integrases, among them two phage integrase site-specific recombinases. The RBG-2 phage genes are not present as an operon, or even on the same scaffolds, and no evidence for a complete phage genome was identified. The RBG-1351 genome encodes a single transposase, homologous to the ISDet1 transposase in *Dehalococcoides mccartyi* 195, and two phage integrases. The RBG-1351 genome contains evidence of a partial phage genome on the largest assembled scaffold, consisting of three phage tail-associated proteins and a phage baseplate assembly protein within a stretch of uncharacterized and hypothetical proteins. There is no evidence for a co-located phage DNA polymerase, capsid proteins, or other phage markers, so the phage is not expected to be functional. Aside from the remnant phage genome, there are four

isolated phage-associated genes: a phage portal protein, a bacteriophage-type DNA polymerase, a DNA-binding protein with homology to bacteriophage lambda, and a phage tail sheath protein. The RBG-9 genome contains eight transposase genes from eight distinct families (IS21, IstA, IstB, ISAca5, 20, IS200, IS3, and ISCc3). Four of the transposases are located in tandem on the longest scaffold, indicating a region where integration may be favoured. The RBG-9 genome also encodes six integrases and two bacteriophage DNA polymerases. There is no other evidence for phage on the genome.

The genomes do not contain any predicted CRISPR regions [41], nor are any of the CRISPR-associated genes encoded, indicating these organisms do not possess this mechanism of phage defense.

Selenocysteine incorporation

Selenocysteine, the 21st naturally occurring amino acid, is utilized by numerous bacteria and archaea, incorporated at a UGA codon that typically codes for a Stop. In bacterial selenocysteine-containing proteins, a selenocysteine insertion sequence (SECIS) forms a hairpin loop immediately downstream of the UGA codon, pausing translation long enough for the alternate coding to be read [42, 43]. All three Chloroflexi genomes encode predicted enzymes for the selenocysteine incorporation pathway, including SelA, an L-seryl-tRNA selenium transferase, SelB, the selenocysteine-specific translation elongation factor, and SelD, a selenide, water dikinase. Based on tRNA scan and BLAST searches, RBG-2 and RBG-1351 lack selC, the selenocysteine tRNA required for interpretation of the TCA codon and incorporation of the selenocysteine onto the nascent peptide chain. The RBG-9 genome contains a more extensive selenate utilization system, encoding not only a complete selenocysteine incorporation pathway (SelABCD), but also the genes associated with direct conversion of selenate to hydrogen selenide and subsequently to selenophosphate or selenocysteine.

Selenocysteine-containing proteins are a functionally diverse group that are typically implicated in redox reactions [44]. RBG-2 encodes nine known selenocysteine-containing protein genes with a UGA stop codon, only one of which, *selD*, has a predicted protein that continues past the UGA stop and a predicted secis element

downstream. RBG-1351's genome contains 5 genes for putative selenocysteine-containing enzymes with a UGA stop codon, four of whose complete predicted protein extends past the UGA. The four putative selenocysteine proteins are SelD, a thiol:disulfide reductase, a glutaredoxin, and a formate dehydrogenase alpha subunit. All four selenocysteine proteins contain predicted SECIS elements just downstream of the stop codon. Interestingly, each potentially non-functional gene has a non-selenocysteine-containing counterpart on the RBG-1351 genome, aside from *selD*. The RBG-9 genome contains 20 genes for putative selenocysteine-containing enzymes with a UGA stop codon, 8 of which extend beyond the UGA stop and contain a predicted SECIS element. For four of the proteins, a non-selenocysteine-containing homolog is present on the genome.

Taken together, it appears as though selenocysteine incorporation is not taking place in RBG-2 or RBG-1351. The absence of *selC* likely renders the systems non-functional, and the only protein expected to require a selenocysteine for proper translation is SelD. In the absence of a functional SelC, SelD is not required. In contrast, it seems plausible that RBG-9 incorporates selenocysteine during translation of eight proteins: SelD, selenoprotein W, thioredoxin, two DsrE, glycine reductase A, heterodisulfide reductase A, and formate dehydrogenase A.

REFERENCES

1. Vignais PM, Colbeau A: **Molecular biology of microbial hydrogenases.** *Current Issues in Molecular Biology* 2004, **6**:159–88.
2. Sorgenfrei O, Linder D, Karas M, Klein A: **A novel very small subunit of a selenium containing [NiFe] hydrogenase of *Methanococcus voltae* is postranslationally processed by cleavage at a defined position.** *European Journal of Biochemistry / FEBS* 1993, **213**:1355–8.
3. Stojanowic A, Mander GJ, Duin EC, Hedderich R: **Physiological role of the F420-non-reducing hydrogenase (Mvh) from *Methanothermobacter marburgensis*.** *Archives of Microbiology* 2003, **180**:194–203.
4. Hedderich R: **Energy-converting [NiFe] hydrogenases from archaea and extremophiles: ancestors of complex I.** *Journal of Bioenergetics and Biomembranes* 2004, **36**:65–75.
5. Sapra R, Bagramyan K, Adams MWW: **A simple energy-conserving system: proton reduction coupled to proton translocation.** *Proceedings of the National Academy of Sciences of the United States of America* 2003, **100**:7545–50.
6. Kanai T, Matsuoka R, Beppu H, Nakajima A, Okada Y, Atomi H, Imanaka T: **Distinct physiological roles of the three [NiFe]-hydrogenase orthologs in the hyperthermophilic archaeon *Thermococcus kodakarensis*.** *Journal of Bacteriology* 2011, **193**:3109–16.
7. Haddock JD, Ferry JG: **Purification and properties of phloroglucinol reductase from *Eubacterium oxidoreducens* G-41.** *The Journal of Biological Chemistry* 1989, **264**:4423–7.
8. Kim S-H, Harzman C, Davis JK, Hutcheson R, Broderick JB, Marsh TL, Tiedje JM: **Genome sequence of *Desulfitobacterium hafniense* DCB-2, a Gram-positive anaerobe capable of dehalogenation and metal reduction.** *BMC Microbiology* 2012, **12**:21.
9. Yu NY, Wagner JR, Laird MR, Melli G, Rey S, Lo R, Dao P, Sahinalp SC, Ester M, Foster LJ, Brinkman FSL: **PSORTb 3.0: improved protein subcellular localization prediction with refined localization subcategories and predictive capabilities for all prokaryotes.** *Bioinformatics* 2010, **26**:1608–15.
10. Mai X, Adams MW: **Purification and characterization of two reversible and ADP-dependent acetyl coenzyme A synthetases from the hyperthermophilic archaeon *Pyrococcus furiosus*.** *Journal of Bacteriology* 1996, **178**:5897–903.
11. Müller V, Grüber G: **ATP synthases: structure, function and evolution of unique energy converters.** *Cellular and Molecular Life Sciences* 2003, **60**:474–94.
12. Müller V, Aufurth S, Rahlfs S: **The Na(+) cycle in *Acetobacterium woodii*: identification and characterization of a Na(+) translocating F(1)F(0)-ATPase with a mixed oligomer of 8 and 16 kDa proteolipids.** *Biochimica et Biophysica Acta* 2001, **1505**:108–20.
13. Rahlfs S, Müller V: **Sequence of subunit a of the Na(+)-translocating F1F0-ATPase of *Acetobacterium woodii*: proposal for residues involved in Na+ binding.** *FEBS Letters* 1999, **453**:35–40.
14. Hesseler M, Bogdanović X, Hidalgo A, Berenguer J, Palm GJ, Hinrichs W, Bornscheuer UT: **Cloning, functional expression, biochemical characterization, and**

structural analysis of a haloalkane dehalogenase from *Plesiocystis pacifica* SIR-1. *Applied Microbiology and Biotechnology* 2011, **91**:1049–60.
15. Reszko AE, Kasumov T, Pierce BA, David F, Hoppel CL, Stanley WC, Des Rosiers C, Brunengraber H: **Assessing the reversibility of the anaplerotic reactions of the propionyl-CoA pathway in heart and liver.** *The Journal of Biological Chemistry* 2003, **278**:34959–65.
16. Einsle O, Niessen H, Abt DJ, Seiffert G, Schink B, Huber R, Messerschmidt A, Kroneck PMH: **Crystallization and preliminary X-ray analysis of the tungsten-dependent acetylene hydratase from *Pelobacter acetylenicus*.** *Acta Crystallographica* 2005, **61**:299–301.
17. Tenbrink F, Schink B, Kroneck PMH: **Exploring the active site of the tungsten, iron-sulfur enzyme acetylene hydratase.** *Journal of Bacteriology* 2011, **193**:1229–36.
18. Singer E, Heidelberg JF, Dhillon A, Edwards KJ: **Metagenomic insights into the dominant Fe(II) oxidizing Zetaproteobacteria from an iron mat at Lō´ihi, Hawai´l.** *Frontiers in Microbiology* 2013, **4**:52.
19. Refojo PN, Sousa FL, Teixeira M, Pereira MM: **The alternative complex III: a different architecture using known building modules.** *Biochimica et Biophysica Acta* 2010, **1797**:1869–76.
20. Pereira MM, Refojo PN, Hreggvidsson GO, Hjorleifsdottir S, Teixeira M: **The alternative complex III from *Rhodothermus marinus* - a prototype of a new family of quinol:electron acceptor oxidoreductases.** *FEBS Letters* 2007, **581**:4831–5.
21. Gao X, Xin Y, Blankenship RE: **Enzymatic activity of the alternative complex III as a menaquinol:auracyanin oxidoreductase in the electron transfer chain of *Chloroflexus aurantiacus*.** *FEBS Letters* 2009, **583**:3275–9.
22. Refojo PN, Teixeira M, Pereira MM: **The Alternative complex III: properties and possible mechanisms for electron transfer and energy conservation.** *Biochimica et Biophysica Acta* 2012, **1817**:1852–9.
23. Refojo PN, Ribeiro MA, Calisto F, Teixeira M, Pereira MM: **Structural composition of alternative complex III: Variations on the same theme.** *Biochimica et Biophysica Acta* 2013.
24. Refojo PN, Teixeira M, Pereira MM: **The alternative complex III of *Rhodothermus marinus* and its structural and functional association with caa3 oxygen reductase.** *Biochimica et Biophysica Acta* 2010, **1797**:1477–82.
25. Kiss H, Nett M, Domin N, Martin K, Maresca JA, Copeland A, Lapidus A, Lucas S, Berry KW, Glavina Del Rio T, Dalin E, Tice H, Pitluck S, Richardson P, Bruce D, Goodwin L, Han C, Detter JC, Schmutz J, Brettin T, Land M, Hauser L, Kyrpides NC, Ivanova N, Göker M, Woyke T, Klenk H-P, Bryant DA: **Complete genome sequence of the filamentous gliding predatory bacterium *Herpetosiphon aurantiacus* type strain (114-95(T)).** *Standards in Genomic Sciences* 2011, **5**:356–70.
26. Pati A, Labutti K, Pukall R, Nolan M, Glavina Del Rio T, Tice H, Cheng J-F, Lucas S, Chen F, Copeland A, Ivanova N, Mavromatis K, Mikhailova N, Pitluck S, Bruce D, Goodwin L, Land M, Hauser L, Chang Y-J, Jeffries CD, Chen A, Palaniappan K, Chain P, Brettin T, Sikorski J, Rohde M, Göker M, Bristow J, Eisen JA, Markowitz V, et al.: **Complete genome sequence of *Sphaerobacter thermophilus* type strain (S 6022).** *Standards in Genomic Sciences* 2010, **2**:49–56.

27. Wu D, Raymond J, Wu M, Chatterji S, Ren Q, Graham JE, Bryant DA, Robb F, Colman A, Tallon LJ, Badger JH, Madupu R, Ward NL, Eisen JA: **Complete genome sequence of the aerobic CO-oxidizing thermophile *Thermomicrobium roseum*.** *PloS One* 2009, **4**:e4207.
28. Garsin DA: **Ethanolamine utilization in bacterial pathogens: roles and regulation.** *Nature Reviews Microbiology* 2010, **8**:290–5.
29. Oremland RS, Stolz JF: **The ecology of arsenic.** *Science* 2003, **300**:939–44.
30. Giloteaux L, Holmes DE, Williams KH, Wrighton KC, Wilkins MJ, Montgomery AP, Smith JA, Orellana R, Thompson CA, Roper TJ, Long PE, Lovley DR: **Characterization and transcription of arsenic respiration and resistance genes during in situ uranium bioremediation.** *The ISME Journal* 2013, **7**:370–83.
31. Qin J, Rosen BP, Zhang Y, Wang G, Franke S, Rensing C: **Arsenic detoxification and evolution of trimethylarsine gas by a microbial arsenite S-adenosylmethionine methyltransferase.** *Proceedings of the National Academy of Sciences of the United States of America* 2006, **103**:2075–80.
32. Weinberg M V, Jenney FE, Cui X, Adams MWW: **Rubrerythrin from the hyperthermophilic archaeon *Pyrococcus furiosus* is a rubredoxin-dependent, iron-containing peroxidase.** *Journal of Bacteriology* 2004, **186**:7888–95.
33. Sztukowska M, Bugno M, Potempa J, Travis J, Kurtz DM: **Role of rubrerythrin in the oxidative stress response of *Porphyromonas gingivalis*.** *Molecular Microbiology* 2002, **44**:479–88.
34. Singh J, Tabita FR: **Roles of RubisCO and the RubisCO-like protein in 5-methylthioadenosine metabolism in the Nonsulfur purple bacterium *Rhodospirillum rubrum*.** *Journal of Bacteriology* 2010, **192**:1324–31.
35. Hanson TE, Tabita FR: **A ribulose-1,5-bisphosphate carboxylase/oxygenase (RubisCO)-like protein from *Chlorobium tepidum* that is involved with sulfur metabolism and the response to oxidative stress.** *Proceedings of the National Academy of Sciences of the United States of America* 2001, **98**:4397–402.
36. Kube M, Beck A, Zinder SH, Kuhl H, Reinhardt R, Adrian L: **Genome sequence of the chlorinated compound-respiring bacterium *Dehalococcoides* species strain CBDB1.** *Nature Biotechnology* 2005, **23**:1269–73.
37. Seshadri R, Adrian L, Fouts DE, Eisen JA, Phillippy AM, Methe BA, Ward NL, Nelson WC, Deboy RT, Khouri HM, Kolonay JF, Dodson RJ, Daugherty SC, Brinkac LM, Sullivan SA, Madupu R, Nelson KE, Kang KH, Impraim M, Tran K, Robinson JM, Forberger HA, Fraser CM, Zinder SH, Heidelberg JF: **Genome sequence of the PCE-dechlorinating bacterium *Dehalococcoides ethenogenes*.** *Science* 2005, **307**:105–8.
38. McMurdie PJ, Behrens SF, Müller JA, Göke J, Ritalahti KM, Wagner R, Goltsman E, Lapidus A, Holmes S, Löffler FE, Spormann AM: **Localized plasticity in the streamlined genomes of vinyl chloride respiring *Dehalococcoides*.** *PLoS Genetics* 2009, **5**:e1000714.
39. Zhuang W-Q, Yi S, Feng X, Zinder SH, Tang YJ, Alvarez-Cohen L: **Selective utilization of exogenous amino acids by *Dehalococcoides ethenogenes* strain 195 and its effects on growth and dechlorination activity.** *Applied and Environmental Microbiology* 2011, **77**:7797–803.
40. Bendtsen JD, Nielsen H, Widdick D, Palmer T, Brunak S: **Prediction of twin-arginine signal peptides.** *BMC Bioinformatics* 2005, **6**:167.

41. Grissa I, Vergnaud G, Pourcel C: **CRISPRFinder: a web tool to identify clustered regularly interspaced short palindromic repeats.** *Nucleic Acids Research* 2007, **35**:W52–7.
42. Zavacki AM, Mansell JB, Chung M, Klimovitsky B, Harney JW, Berry MJ: **Coupled tRNA(Sec)-dependent assembly of the selenocysteine decoding apparatus.** *Molecular Cell* 2003, **11**:773–81.
43. Donovan J, Copeland PR: **Evolutionary history of selenocysteine incorporation from the perspective of SECIS binding proteins.** *BMC Evolutionary Biology* 2009, **9**:229.
44. Zhang Y, Romero H, Salinas G, Gladyshev VN: **Dynamic evolution of selenocysteine utilization in bacteria: a balance between selenoprotein loss and evolution of selenocysteine from redox active cysteine residues.** *Genome Biology* 2006, **7**:R94.
